# A network meta-analysis on the efficacy of sixteen targeted drugs in combination with chemotherapy for treatment of advanced/metastatic colorectal cancer

**DOI:** 10.18632/oncotarget.12994

**Published:** 2016-10-31

**Authors:** Dan-Zeng Ba-Sang, Zi-Wen Long, Hao Teng, Xu-Peng Zhao, Jian Qiu, Ming-Shan Li

**Affiliations:** ^1^ Department of Oncology, Shigatse People's Hospital, Shigatse 857000, Tibet, P. R. China; ^2^ Department of Gastric Cancer and Soft-Tissue Sarcoma Surgery, Fudan university Shanghai Cancer Center, Shanghai 200032, P. R. China; ^3^ Department of Oncology, Shanghai Medical College, Fudan University, Shanghai 200032, P. R. China

**Keywords:** colorectal cancer, targeted drug, chemotherapy, randomized controlled trial, network meta-analysis

## Abstract

**Objective:**

A network meta-analysis was conducted comparing the short-term efficacies of 16 targeted drugs in combination with chemotherapy for treatment of advanced/metastatic colorectal cancer (CRC).

**Results:**

Twenty-seven RCTs were ultimately incorporated into this network meta-analysis. Compared with chemotherapy alone, bevacizumab + chemotherapy, panitumumab + chemotherapy and conatumumab + chemotherapy had higher PR rate. Bevacizumab + chemotherapy, cetuximab + chemotherapy, panitumumab + chemotherapy, trebananib + chemotherapy and conatumumab + chemotherapy had higher ORR rate in comparison to chemotherapy alone. Furthermore, bevacizumab + chemotherapy had higher DCR rate than chemotherapy alone. The results of our cluster analysis showed that chemotherapy combined with bevacizumab, cetuximab, panitumumab, conatumumab, ganitumab, or brivanib + cetuximab had better efficacies for the treatment of advanced/metastatic CRC in comparison to chemotherapy alone.

**Materials and Methods:**

Electronic databases were comprehensively searched for potential and related randomized controlled trials (RCTs). Direct and indirect evidence were incorporated for evaluation of stable disease (SD), progressive disease (PD), complete response (CR), partial response (PR), disease control rate (DCR) and overall response ratio (ORR) by calculating odds ratio (OR) and 95% confidence intervals (CI), and using the surface under the cumulative ranking curve (SUCRA).

**Conclusions:**

These results indicated that bevacizumab + chemotherapy, panitumumab + chemotherapy, conatumumab + chemotherapy and brivanib + cetuximab + chemotherapy may have better efficacies for the treatment of advanced/metastatic CRC.

## INTRODUCTION

Colorectal Cancer (CRC) is a malignant disease of worldwide concern mainly affecting the elderly with a general onset age of 66 years old [1]. According to the World Cancer Report, there were 694,000 CRC deaths in 2012 [2]. The survival rate for CRC in a 5-year period increased from 51% to 65% due to developments in medical science and treatment methods, but fatal metastatic CRC remains is significant threat to public health [3]. Currently, surgical resection combined with chemotherapy is the frontline treatment for CRC; however, chemotherapy remains flawed, as the desired therapeutic effects are achieved in only 30% of patients. This might be due to the fact that conventional chemotherapy drugs are toxic to both tumor and normal tissues, and therefore cause a variety of side effects, including neutropenia, anemia, stomach poisoning disease, hematopoietic disorders and poisoning stem cells [4]. The application of drugs specifically targeting tumor tissue would reduce the interference to normal tissues and decrease side effects [5].

Current targeted drugs can be roughly divided into the following categories: (1) drugs that inhibit VEGF-related factors to reduce angiopoiesis, including bevacizumab [6], cediranib [7], axitinib [8], and sorafenib [9]; (2) drugs that inhibit EGFR-related factors to control tumor cell proliferation and differentiation, including cetuximab [10], panitumumab [11], and gefitinib [12]; (3) drugs that inhibit angiopoietin, including sunitinib [13], celecoxib [14], and trebananib [15]; and (4) drugs that inhibit tumor proliferation though other relevant pathways, e.g. conatumumab causes apoptosis of cancer cells by targeting DR5 [16] and ganitumab inhibits tumor cell proliferation by inhibiting IGF signaling [17]. Combined treatment with anticancer drugs from different chemotherapy regimens is also commonly used. A previous study demonstrated that sunitinib-based combination was associated with more toxicity, and combination therapy with sunitinib plus mFOLFOX6 was not recommended in patients with metastatic colorectal cancer [18]. Moreover, the combination use of sorafenib in combination with mFOLFOX6 chemotherapy regimen was not preferable in metastatic colorectal cancer [19]. Despite the abundant literature submitted, no comprehensive literature regarding the optimal strategy on combined targeted drugs with chemotherapy in colorectal cancer was available.

The study of targeted drugs is based on clinical data from long-term evaluation of patients, for which the selection of methodology is an important factor affecting research conclusions [20]. Meta-analysis is a commonly used clinical analytical tool; with sufficient data its conclusions are normally reliable. However, traditional meta-analysis is often applied to paired comparison, in which case, the conclusions often less accurate [20]. Network meta-analysis can make up this deficiency, implementing a direct and indirect quantified comparison of different treatments with similar disease interventions and then selecting the optimal treatment [21]. This study integrates the research data of 16 targeted drugs combined with chemotherapy for the treatment of advanced/metastatic CRC, attempting to uncover the best treatment options for advanced/metastatic CRC through network meta-analysis.

## RESULTS

### Baseline characteristics of included study

**Figure 1:**
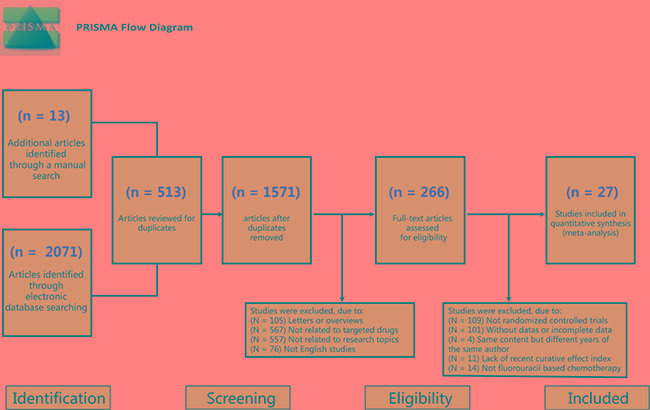
Flow chart showing literature search and study selection Twenty-seven randomized controlled trials that met the inclusion criteria were included in this network meta-analysis.

**Figure 2:**
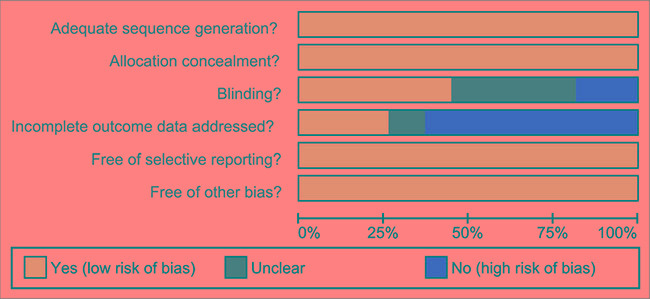
Quality assessment diagram of 27 included studies in this network meta-analysis

Our electronic database searches provided 2084 candidate studies. After reviewing the titles and abstracts, we excluded 513 studies as duplicates, 105 as letters or summaries, 76 as non-English studies, 557 as unrelated to advanced/metastatic CRC, and 567 as non-targeted drug studies. Upon further assessment of the remaining 266 articles, we excluded 109 studies of non-RCTs, 101 studies without data resources or incomplete documentation, 4 articles by the same author with the same content in a different document, 11 articles with incomplete documentation on short-term efficacy, and 14 studies of non-fluorouracil-based chemotherapy. Eventually, 27 RCTs were found eligible for this network meta-analysis [18, 19, 22–46] and the PRISMA screening flow chart (see Figure 1). Together these 27 RCTs included 9031 patients with advanced/metastatic CRC, including 2584 cases treated with chemotherapy alone (19 studies), 1936 cases treated with bevacizumab + chemotherapy (15 studies), 1598 cases treated with cetuximab + chemotherapy (7 studies), 632 cases treated with panitumumab + chemotherapy (3 studies), 482 cases treated with sunitinib + chemotherapy (2 studies), 853 cases treated with cediranib + chemotherapy (2 study), 19 cases treated with celecoxib + chemotherapy (1 study ) and 97 cases treated with sorafenib + chemotherapy (1 study), 133 cases treated with axitinib + chemotherapy (3 studies), 95 cases treated with trebananib + chemotherapy (1 study), 51 cases treated with conatumumab + chemotherapy (1 study), 52 cases treated with ganitumab + chemotherapy (1 study), 137 cases treated with panitumumab + bevacizumab + chemotherapy (1 study ), 376 cases treated with brivanib + cetuximab + chemotherapy (1 study), 82 cases treated with axitinib + bevacizumab + chemotherapy (2 studies), and 123 cases treated with cetuximab + bevacizumab + chemotherapy (1 study). The included studies were published between 2006 and 2015. Baseline characteristics of the included studies were summarized in Supplementary Table S1. Cochrane systematic bias for all included studies was shown in Figure 2.

### Pairwise meta-analysis

We carried out direct paired comparisons for the short-term efficacies of 16 treatments on advanced/metastatic CRC patients. Cetuximab + chemotherapy, sunitinib + chemotherapy, sorafenib + chemotherapy, panitumumab + bevacizumab + chemotherapy for advanced/metastatic CRC patients had lower SD rate than chemotherapy alone (all *P* < 0.05). The PD rate of bevacizumab + chemotherapy for advanced/metastatic CRC patients was relatively lower than chemotherapy alone (OR = 0.533, *P* = 0.004). Bevacizumab + chemotherapy and cetuximab + chemotherapy had higher PR rate than chemotherapy alone (both *P* *<* 0.05). In terms of ORR rate, bevacizumab + chemotherapy, cetuximab + chemotherapy, panitumumab + chemotherapy were better than chemotherapy alone (all *P* *<* 0.05). However, the CR and DCR rates of all targeted drugs combined with chemotherapy were not significantly different with chemotherapy alone (all *P* > 0.05) (Supplementary Table S2).

### Forest plots for the efficacy of 16 targeted drugs combined with chemotherapy

**Figure 3:**
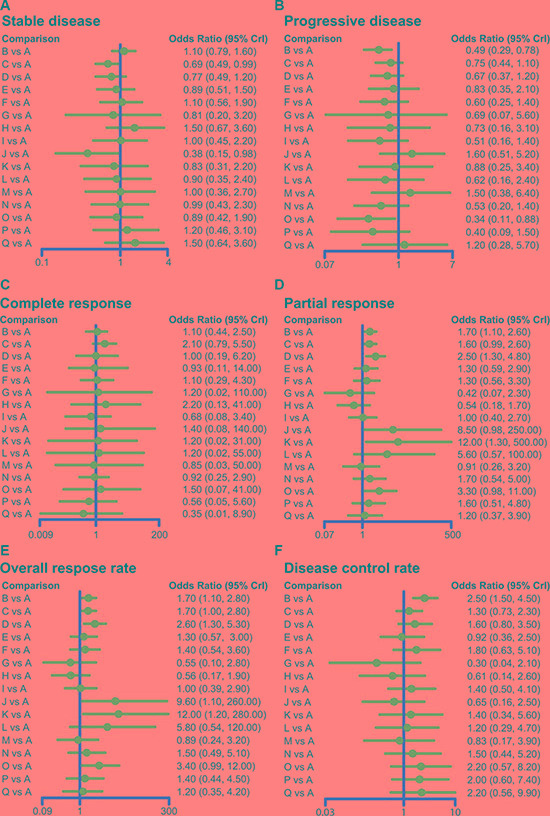
Forest plots for the efficacy of 16 targeted drugs combined with chemotherapy in the treatment of advanced/metastatic colorectal cancer The black solid lines represent the confidence intervals for summary odds ratios for each comparison. (A: chemotherapy; B: bevacizumab + chemotherapy; C: cetuximab + chemotherapy; D: panitumumab + chemotherapy; E: sunitinib + chemotherapy; F: cediranib + chemotherapy; G: celecoxib + chemotherapy; H: sorafenib + chemotherapy; I: axitinib + chemotherapy; J: trebananib + chemotherapy; K: conatumumab + chemotherapy; L: ganitumab + chemotherapy; M: gefitinib + chemotherapy; N: panitumumab + bevacizumab + chemotherapy; O: brivanib + cetuximab + chemotherapy; P: axitinib + bevacizumab + chemotherapy; Q: cetuximab + bevacizumab + chemotherapy).

The relative relationships to direct and indirect short-term efficacy of the 16 targeted drugs combined with chemotherapy for advanced/metastatic CRC patients showed that: (1) The SD rates of cetuximab + chemotherapy and trebananib + chemotherapy were relatively lower than chemotherapy alone, while the SD rates of other targeted drugs combined with chemotherapy showed no statistically significant difference with chemotherapy alone. (2) The PD rates of bevacizumab + chemotherapy and brivanib + cetuximab + chemotherapy were relatively lower than that chemotherapy alone. No difference was also found between other targeted drugs combined with chemotherapy and chemotherapy alone. (3) The CR rates of the 16 targeted drugs combined with chemotherapy showed no obvious difference with that of chemotherapy alone. (4) The PR rates of bevacizumab + chemotherapy, panitumumab + chemotherapy, conatumumab + chemotherapy were higher than that of chemotherapy alone, while other targeted drugs combined with chemotherapy showed no difference with chemotherapy alone. (5) The ORR rates of bevacizumab + chemotherapy, cetuximab + chemotherapy, panitumumab + chemotherapy, trebananib + chemotherapy, and conatumumab + chemotherapy were higher than that of chemotherapy alone, while other targeted drugs combined with chemotherapy showed no significant difference with the efficacy of chemotherapy alone. (6) The DCR rates of bevacizumab + chemotherapy was better than that of chemotherapy alone, while the efficacy of other targeted drugs in combination with chemotherapy had no statistical difference when compared to the efficacy of chemotherapy (Figure 3, Supplementary Table S3).

### Evidence network of network meta-analysis

**Figure 4:**
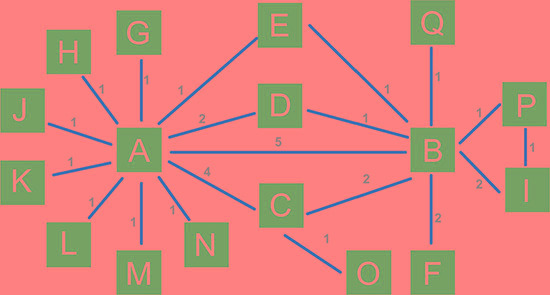
Evidence network plot of 17 treatment regimens which were included in this network meta-analysis (A: chemotherapy; B: bevacizumab + chemotherapy; C: cetuximab + chemotherapy; D: panitumumab + chemotherapy; E: sunitinib + chemotherapy; F: cediranib + chemotherapy; G: celecoxib + chemotherapy; H: sorafenib + chemotherapy; I: axitinib + chemotherapy; J: trebananib + chemotherapy; K: conatumumab + chemotherapy; L: ganitumab + chemotherapy; M: gefitinib + chemotherapy; N: panitumumab + bevacizumab + chemotherapy; O: brivanib + cetuximab + chemotherapy; P: axitinib + bevacizumab + chemotherapy; Q: cetuximab + bevacizumab + chemotherapy).

A total of 17 kinds of treatment regimens were included in the current study (A: chemotherapy; B: bevacizumab + chemotherapy; C: celecoxib + chemotherapy; D: panitumumab + chemotherapy; E: sunitinib + chemotherapy; F: cediranib + chemotherapy; H: sorafenib + chemotherapy; I: axitinib + chemotherapy; J: trebananib + chemotherapy; K: conatumumab + chemotherapy; L: ganitumab + chemotherapy; M: gefitinib + chemotherapy; N: panitumumab + bevacizumab + chemotherapy; O: brivanib + cetuximab + chemotherapy; P: axitinib + bevacizumab + chemotherapy; Q: cetuximab + bevacizumab + chemotherapy). The network plot displays the short-term effects of the 17 treatment regimens on patients with advanced/metastatic CRC, and the data for every direct comparison of two treatment regimens are included in Figure 4.

### Inconsistency test of network meta-analysis

We performed an inconsistency test on the 27 included studies which constituted 4 closed loops that formed a ring of research studies (5 studies of chemotherapy vs. bevacizumab + chemotherapy; 4 studies of chemotherapy vs. cetuximab + chemotherapy; 2 studies of bevacizumab + chemotherapy vs. cetuximab + chemotherapy; 5 studies of chemotherapy vs. bevacizumab + chemotherapy; 2 studies of chemotherapy + chemotherapy vs. panitumumab + chemotherapy, 1 study of bevacizumab + chemotherapy vs. panitumumab + chemotherapy; 5 studies of chemotherapy vs. bevacizumab + chemotherapy; 1 study of chemotherapy vs. sunitinib + chemotherapy; 1 study of bevacizumab + chemotherapy vs. sunitinib + chemotherapy; 2 studies of bevacizumab + chemotherapy vs. axitinib + chemotherapy; 1 study of bevacizumab + chemotherapy vs. axitinib + bevacizumab + chemotherapy, 1 study of axitinib + chemotherapy vs. axitinib + bevacizumab + chemotherapy). The results showed no inconsistencies among the studies in terms of SD, PD, CR, PR, DCR, or ORR (all *P* > 0.05) (Table 1). Therefore, the consistency model is applicable.

**Table 1:** OR values and *P* values of direct and indirect pairwise comparisons of seventeen treatment modalities under six endpoint outcomes

Pairwise comparisons	Direct OR values						Indirect OR values						*P* values					
	SD	PD	CR	PR	ORR	DCR	SD	PD	CR	PR	ORR	DCR	SD	PD	CR	PR	ORR	DCR
A VS B	−0.017	−0.730	0.405	0.603	0.690	0.982	0.275	−0.432	−0.501	0.355	0.313	0.774	0.334	0.488	0.268	0.556	0.389	0.681
A VS C	−0.362	−0.187	0.334	0.526	0.549	0.250	−0.366	−0.562	1.385	0.297	0.432	0.185	0.993	0.444	0.246	0.653	0.828	0.915
A VS D	−0.171	−0.336	0.006	0.798	0.802	0.386	−0.608	−0.534	0.136	1.227	1.327	0.785	0.356	0.741	0.942	0.538	0.472	0.595
A VS E	−0.032	−0.278	−0.010	−0.051	−0.063	−0.172	−0.319	0.553	0.124	0.682	0.723	0.070	0.577	0.501	0.948	0.300	0.296	0.795
B VS C	−0.474	0.136	1.022	−0.157	−0.087	−0.696	−0.471	0.511	−0.029	0.072	0.030	−0.631	0.993	0.444	0.246	0.653	0.829	0.915
B VS D	−0.668	0.100	−0.001	0.682	0.734	−0.160	−0.231	0.298	−0.130	0.253	0.209	−0.560	0.356	0.741	0.942	0.538	0.472	0.595
B VS E	−0.400	1.109	−0.011	0.116	0.115	−0.843	−0.113	0.278	−0.145	−0.617	−0.671	−1.085	0.576	0.501	0.948	0.300	0.296	0.795
B VS F	−0.057	0.157	0.023	−0.215	−0.217	−0.350	0.202	1.124	−0.516	−1.099	−1.219	−1.131	0.998	0.994	0.999	0.993	0.991	0.994
B VS I	0.032	0.189	−0.376	−0.384	−0.414	−0.376	−0.611	−0.537	−0.693	−0.803	−0.870	−1.425	0.463	0.558	0.881	0.674	0.657	0.313
B VS P	−0.167	−0.511	−0.668	−0.213	−0.400	−0.672	0.475	0.215	−0.352	0.206	0.056	0.376	0.464	0.558	0.881	0.674	0.657	0.313
B VS Q	0.327	0.849	−0.693	−0.349	−0.380	−0.117	−0.613	−0.827	−0.754	−0.210	−0.298	−1.186	0.994	0.993	1.000	0.999	1.000	0.994
C VS O	0.258	−0.802	−0.005	0.701	0.701	0.524	1.164	2.184	−4.997	−4.003	−4.214	−2.238	0.994	0.987	0.992	0.972	0.970	0.985
I VS P	0.443	0.026	0.025	0.590	0.470	0.752	−0.199	−0.700	−0.292	0.171	0.014	−0.296	0.463	0.558	0.881	0.674	0.657	0.313

Notes: SD = stable disease; PD = progressive disease; CR = complete response; PR = partial response; ORR = overall response rate; DCR = disease control rate; ORR = CR+PR; DCR = SD+CR+PR; OR = odd ratios; A = chemotherapy; B = bevacizumab+chemotherapy; C = cetuximab+chemotherapy; D = panitumumab+chemotherapy; E = sunitinib+chemotherapy; F = cediranib+chemotherapy; I = axitinib+chemotherapy; O = brivanib+cetuximab+chemotherapy; P = axitinib+bevacizumab+chemotherapy; Q = cetuximab+bevacizumab+chemotherapy.

### Surface under the cumulative ranking curves (SUCRA)

The SUCRA values of the 16 treatment regimens for advanced/metastatic CRC patients are summarized in Table 2. The SUCRA curves indicated that: (1) the combined therapies of cetuximab + bevacizumab + chemotherapy, sorafenib + chemotherapy, bevacizumab + chemotherapy, and axitinib + bevacizumab + chemotherapy had lower SD rates for the treatment of advanced/metastatic CRC treatment; the SUCRA values of which were 0.81, 0.79, 0.65, and 0.65, respectively; (2) the combined therapies of brivanib + cetuximab + chemotherapy, axitinib + bevacizumab + chemotherapy, bevacizumab + chemotherapy had lower PD rates for the treatment of advanced/metastatic CRC, the SUCRA values of which were 0.86, 0.78, and 0.72, respectively; (3) the combined therapies of cetuximab + chemotherapy, brivanib + cetuximab + chemotherapy, and sorafenib + chemotherapy had higher CR rates for the treatment of advanced/metastatic CRC, the SUCRA values of which were 0.74, 0.69, and 0.66, respectively; (4) the combined therapies of conatumumab + chemotherapy, trebananib + chemotherapy, ganitumab + chemotherapy had higher PR rates for the treatment of advanced/metastatic CRC, the SUCRA values of which were 0.90, 0.86, and 0.80, respectively; (5) the combined therapies of conatumumab + chemotherapy, trebananib + chemotherapy, and ganitumab + chemotherapy had higher ORR rates for the treatment of advanced/metastatic CRC, the SUCRA values of which were 0.91, 0.88, and 0.79, respectively; and (6) the combined therapies of bevacizumab + chemotherapy, cetuximab + bevacizumab + chemotherapy, brivanib + cetuximab + chemotherapy had higher DCR rates for the treatment of advanced/metastatic CRC, the SUCRA value of which were 0.86, 0.74, and 0.73, respectively.

**Table 2:** The results of surface under the cumulative ranking curve of 16 targeted drugs

Code	Treatment regimen	SD	PD	CR	PR	ORR	DCR
A	Chemotherapy	0.55	0.27	0.46	0.26	0.22	0.34
B	Bevacizumab + Chemotherapy	0.65	0.72	0.53	0.56	0.57	0.86
C	Cetuximab + Chemotherapy	0.24	0.46	0.74	0.53	0.55	0.49
D	Panitumumab + Chemotherapy	0.33	0.52	0.53	0.72	0.72	0.62
E	Sunitinib + Chemotherapy	0.43	0.42	0.51	0.41	0.40	0.34
F	Cediranib + Chemotherapy	0.59	0.57	0.51	0.43	0.44	0.66
G	Celecoxib + Chemotherapy	0.43	0.55	0.51	0.10	0.16	0.13
H	Sorafenib + Chemotherapy	0.79	0.50	0.66	0.12	0.13	0.24
I	Axitinib + Chemotherapy	0.55	0.65	0.36	0.30	0.30	0.54
J	Trebananib + Chemotherapy	0.06	0.14	0.50	0.86	0.88	0.22
K	Conatumumab + Chemotherapy	0.42	0.38	0.47	0.90	0.91	0.54
L	Ganitumab + Chemotherapy	0.49	0.59	0.50	0.80	0.79	0.47
M	Gefitinib + Chemotherapy	0.53	0.18	0.54	0.28	0.28	0.31
N	Panitumumab + Bevacizumab + Chemotherapy	0.53	0.66	0.45	0.54	0.49	0.57
O	Brivanib + Cetuximab + Chemotherapy	0.46	0.86	0.69	0.77	0.77	0.73
P	Axitinib + Bevacizumab + Chemotherapy	0.65	0.78	0.32	0.51	0.44	0.71
Q	Cetuximab + Bevacizumab + Chemotherapy	0.81	0.27	0.25	0.37	0.38	0.74

Notes: SD = stable disease; PD = progressive disease; CR = complete response; PR = partial response; ORR = overall response rate; DCR = disease control rate; ORR = CR + PR; DCR = SD + CR + PR.

### Cluster analysis

**Figure 5:**
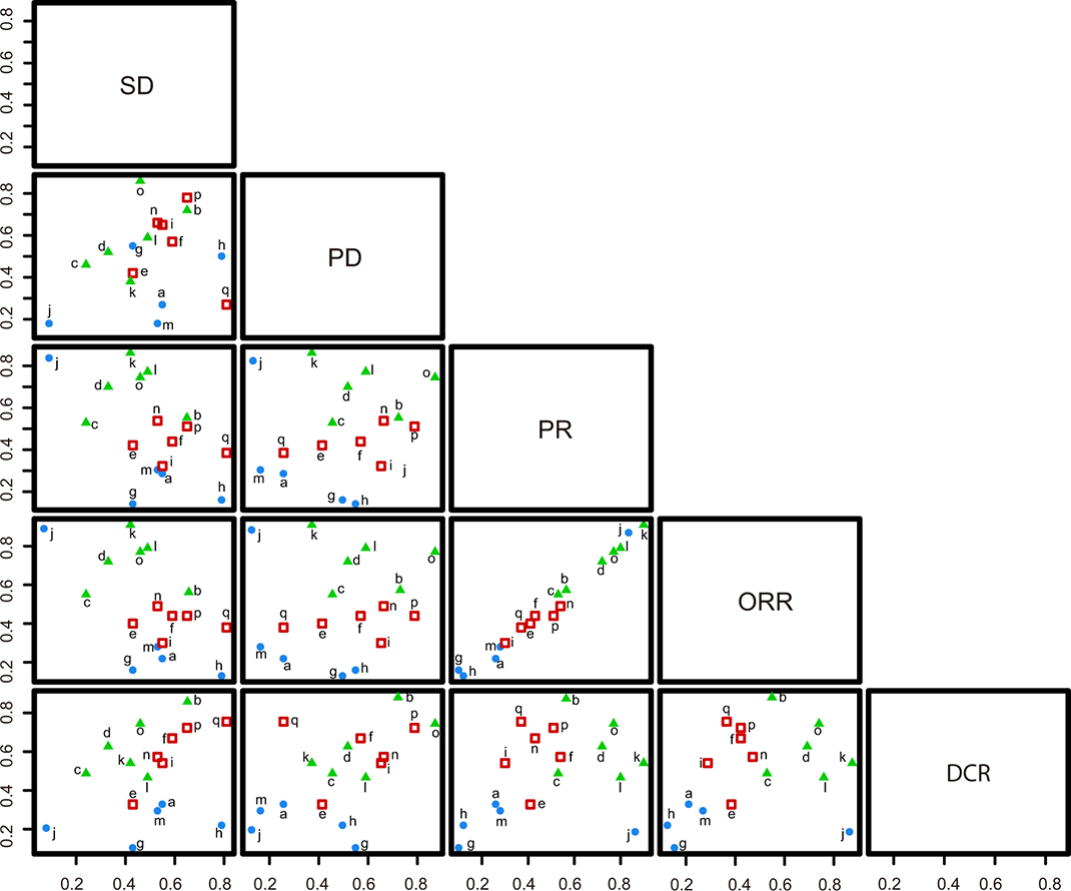
Clustered ranking plots based on SUCRA values of the SD, PD, PR, ORR and DCR rates of 17 treatment regimens in the treatment of advanced/metastatic colorectal cancer (a: chemotherapy; b: bevacizumab + chemotherapy; c: cetuximab + chemotherapy; d: panitumumab + chemotherapy; e: sunitinib + chemotherapy; f: cediranib + chemotherapy; g: celecoxib + chemotherapy; h: sorafenib + chemotherapy; i = axitinib + chemotherapy; j = trebananib + chemotherapy; k = conatumumab + chemotherapy; l = ganitumab + chemotherapy; m = gefitinib + chemotherapy; n = panitumumab + bevacizumab + chemotherapy; o = brivanib + cetuximab + chemotherapy; p = axitinib + bevacizumab + chemotherapy; q = cetuximab + bevacizumab + chemotherapy).

Cluster analysis of SUCRA values based on SD vs. PD, SD vs. PR, SD vs. ORR, SD vs. DCR shows that the combined therapies of bevacizumab + chemotherapy, cetuximab + chemotherapy, panitumumab + chemotherapy, conatumumab + chemotherapy, ganitumab + chemotherapy, brivanib + cetuximab + chemotherapy were relatively good for the treatment of advanced/metastatic CRC. SUCRA value results of cluster analysis based on ORR vs. DCR showed that: the combined therapies of bevacizumab + chemotherapy, cetuximab + chemotherapy, panitumumab + chemotherapy, conatumumab + chemotherapy, ganitumab + chemotherapy and brivanib + cetuximab + chemotherapy were relatively good in efficacy for the treatment of advanced/metastatic CRC (Figure 5).

## DISCUSSION

Integrating currently available data, current network meta-analysis of targeted drug therapy proved that the efficacies of the combined therapies were better than the efficacy of chemotherapy alone. Our results showed that the efficacy of bevacizumab + chemotherapy, sorafenib + chemotherapy, panitumumab + chemotherapy, trebananib + chemotherapy and conatumumab + chemotherapy were more prominent among all the combined chemotherapies. Evidence supported that monoclonal antibody-based treatment of cancer has been proved as one of the most successful therapeutic strategies for both hematologic malignancies and solid tumors [47, 48]. Several antibodies were included as targeted drugs in current study, including bevacizumab and conatumumab. Bevacizumab is an anti-VEGF monoclonal antibody, which has a good efficacy and can prolong the survival time of advanced/metastatic CRC patients [23–24]. Sorafenib is a multi-targeted tyrosine kinase, which can control angiogenesis by inhibiting the VEGF receptor, effective for the treatment of tumors [29]. Besides, the combined therapy of trebananib with anti-VEGF drugs, such as sorafenib, bevacizumab or motesanib, presented with apparently less toxicity and more effective in inhibiting the proliferation of tumors cells [49, 50]. Conatumumab is a monoclonal antibody which targets at DR5 and thus mediates the apoptosis of tumor cells with low toxicity to normal cells [19, 27, 51]. Conatumumab is a relevant factor that can cause apoptosis of the tumor cells and can be applied with combination of 5-fluorouracil, leucovorin and irinotecan. The combination of these agents can reduce the toxic effects of the body and thus distinctively mediate the apoptosis of tumor cells [52]. Consistent with these findings, the results of this study showed that the efficacies of bevacizumab + chemotherapy, panitumumab + chemotherapy, conatumumab + chemotherapy and trebananib + chemotherapy were relatively more significant than the efficacy of chemotherapy alone. Panitumumab is a complex which inhibits the expression of downstream molecules indirectly by binding to EGFR, so as to inhibit the proliferation and differentiation of tumor cells [24]. Trebananib is a peptide hybrid protein, which can deter the formation of tumors by interfering with the binding of angiopoietin-1 and angiopoietin-2 to Tie2 receptor [28].

Additionally, our study found that the efficacy of brivanib + celecoxib + chemotherapy is distinctively better than the efficacies of cetuximab + chemotherapy and sorafenib + chemotherapy. A perspective study has demonstrated that both brivanib and cetuximab inhibit the VEGF signaling pathway, so as to cooperate with each other in inhibiting tumor cell-induced angiogenesis [29, 30]. Greening *et al.* also pointed out that the efficacy of brivanib and cetuximab was significantly better than the efficacy of cetuximab and cetuximab combined with irinotecan [53]. However, no direct report proves that the efficacy of combined brivanib and cetuximab therapy is better than the efficacy of Sorafenib therapy. In addition, the combined therapy of brivanib and cetuximab has been reported to produce a certain toxicity which has a negative impact on the overall survival of patients [28], which may explain why the efficiency of combined therapy of brivanib and cetuximab was inferior to that of trebananib. However, our results revealed that the efficacy of brivanib + celecoxib + chemotherapy is distinctively better than the efficacy of trebananib + chemotherapy. But further research is needed to discover the advantages and disadvantages of both therapies. From pharmacodynamic perspective, the advantages of the combined therapy of brivanib and cetuximab may occur because it not only inhibits VEGF-induced angiogenesis of tumor cells, but also inhibits EGFR signaling pathways in parallel to further inhibit the proliferation of tumor cells [54].

Further analysis of the results of SUCRA revealed that the efficacies of trebananib + chemotherapy, conatumumab + chemotherapy, and ganitumab + chemotherapy are relatively higher in regard to PR and ORR. This may due to the fact that the included documents and data are relatively limited, which may result in a deviation of the results. Another reason may be the exclusion criteria based on the analysis of pharmacological effects. As mentioned earlier, trebananib is effective in inhibiting the angiogenesis-related pathway in tumor cells [49, 50]. Ganitumab inhibits the IGF signaling pathway by binding to IGF1R and thus inhibits proliferation of tumor cells. Although limited data on the efficacy of ganitumab combined with other drugs exists, clinical research shows that combined therapy of panitumumab and rilotumumab is much more efficacious than ganitumab therapy alone [55]. Thus, the actual efficacy of ganitumab therapy requires further study.

Due to the limited references and data, this study has integrated the current number of chemotherapy drugs which are compared and analyzed without yielding obvious conclusions on the ideal drugs for the progression or metastasis of advanced/metastatic CRC. Deviation occurs in network meta-analysis when the data are insufficient for the evaluation [56, 57]. Correction of this deviation relies on the optimization of the new algorithm and more clinical and basic research data for further correction and investigation. In summary, the results of this network meta-analysis suggest that bevacizumab + chemotherapy, panitumumab + chemotherapy, conatumumab + chemotherapy and brivanib + cetuximab + chemotherapy may have better efficacy for the treatment of advanced/metastatic CRC, which provide evidence toward further development of more effective treatment of advanced/metastatic CRC and inspiration for further study.

## MATERIALS AND METHODS

### Search strategy

PubMed, Embase, Cochrane central register of controlled trials (CENTRAL), and other English language databases were searched from the inception of each database through September 2015. Searches were conducted using the keywords and combined words: colorectal neoplasms, colorectal tumor, colorectal cancer, colorectal carcinomas, bevacizumab, cetuximab, panitumumab, sunitinib, cediranib, celecoxib, sorafenib, axitinib, trebananib, conatumumab, ganitumab, gefitinib, randomized controlled trial, and chemotherapy.

### Inclusion and exclusion criteria

The inclusion criteria were: (1) study design: randomized controlled trial (RCT); (2) study subjects: advanced/metastatic CRC patients; (3) treatment regimens: targeted drugs combined with chemotherapy; (4) end outcomes: stable disease (SD), progressive disease (PD), complete response (CR), partial response (PR), disease control rate (DCR), and overall response ratio (ORR). The exclusion criteria were: (1) non-English reference; (2) non-RCTs; (3) duplicated publications from the same author with different interventions; (4) review and letters; (5) non-human research subjects; (6) documents with insufficient end outcomes; (7) non-fluorouracil-based chemotherapy study.

### Data extraction

Two reviewers extracted data from the enrolled studies using a specifically designed form. Additionally, a third reviewer was consulted if agreement could not be reached between these two reviewers. Two or more researchers reviewed the RCTs according to Cochrane risk of bias assessment tools [58], which includes six domains: adequate sequence generation, allocation concealment, blinding, incomplete outcome data addressed, free of selective reporting, and free of other biases.

### Statistical analysis

Traditional pairwise meta-analyses were performed to directly compared the short-term efficacies (CR, PR, ORR, SD, PD and DCR) of 16 targeted drugs (Bevacizumab, Cetuximab, Panitumumab, Sunitinib, Cediranib, Celecoxib, Sorafenib, Axitinib, Trebananib, Conatumumab, Ganitumab, Gefitinib, Panitumumab plus Bevacizumab, Brivanib plus Cetuximab, Axitinib plus Bevacizumab, Cetuximab plus Bevacizumab) in combination with chemotherapy for the treatment of advanced/metastatic CRC. Bayesian network meta-analyses were performed to indirectly compare CR, PR, ORR, SD, PD and DCR of 16 targeted drugs in combination with chemotherapy for the treatment of advanced/metastatic CRC. Fixed-effect and random-effects model proposed by Woods and co-workers was applied. The node-splitting plot statistic was used to assess the extent of inconsistency, and ontology consistent model was applied if *P* > 0.05. We checked and confirmed convergence and lack of auto correlation after four chains and a 20,000-simulation burn-in phase. Direct probability statements were derived from an additional 50,000-simulation phase [59]. The surface under the cumulative ranking curve (SUCRA) was used to evaluate the efficacies of 16 targeted drugs in combination with chemotherapy for the treatment of advanced/metastatic CRC [60]. The plot of a network of interventions is a visual representation of the evidence base and offers a concise description of its characteristics according to the SUCRA values, the larger the SUCRA value, the better the rank of the intervention [61]. The network meta-analyses were performed using Stata 13.1 (Stata Corp, College Station, TX, USA) software and R (V.3.1.2) package gemtc (V.0.6), 13 14 along with the Markov Chain Monte Carlo engine Open BUGS (V.3.4.0).

## SUPPLEMENTARY MATERIALS FIGURES AND TABLES








