# Supplementary material for: A network meta-analysis on the efficacy of sixteen targeted drugs in combination with chemotherapy for treatment of advanced/metastatic colorectal cancer
Source: Oncotarget. 2016 Oct 31;7(51):84468–79. doi: 10.18632/oncotarget.12994 (PMC5356673; doi:10.18632/oncotarget.12994)
Supplement: Supplementary file 1 [file oncotarget-07-84468-s001.pdf]

A network meta-analysis on the efficacy of sixteen targeted drugs in combination with chemotherapy for treatment of advanced/metastatic colorectal cancer

Supplementary Materials

Supplementary Table S1: Main characteristics and methodological quality of eligibly studies. See Supplementary_Table_S1

Supplementary Table S2: Estimated OR and 95% CI produced by random effects pairwise meta-analysis for efficacy events in metastatic/advanced colorectal cancer patients. See Supplementary_Table_S2

Supplementary Table S3: Odds ratios and 95% confidence intervals of 17 treatment modalities under six end indicators according to the network meta-analysis. See Supplementary_Table_S3
