# Supplementary material for: A network meta-analysis on the efficacy of sixteen targeted drugs in combination with chemotherapy for treatment of advanced/metastatic colorectal cancer
Source: Oncotarget. 2016 Oct 31;7(51):84468–79. doi: 10.18632/oncotarget.12994 (PMC5356673; doi:10.18632/oncotarget.12994)
Supplement: Supplementary file 2 [file oncotarget-07-84468-s002.docx]

**Table S1:** Main characteristics and methodological quality of eligibly studies.

| **Author** | **Year** | **Country** | **Patients numbers** | **Population** | **Targeted drugs** | | **Study Design** | **Follow-up**  **(months)** | **Gender(M/F)** | |
| --- | --- | --- | --- | --- | --- | --- | --- | --- | --- | --- |
|  |  |  |  |  | **Treat 1** | **Treat 2** |  |  | **Treat 1** | **Treat 2** |
| Hecht JR | 2015 | USA | 191 | ITT | Bevacizumab+C | Sunitinib+C | RCT | 24-47 | 62/33 | 61/35 |
| Hecht JR | 2015 | USA | 182 | ITT | Bevacizumab+C | Panitumumab+C | RCT | 0-58 | 58/33 | 62/29 |
| Xie S | 2014 | China | 292 | ITT | Panitumumab+Bevacizumab+C | C | RCT | NR | 81/56 | 98/57 |
| Peeters M-a | 2014 | Belgium | 597 | WT KRAS | Panitumumab+C | C | RCT | 1-206 | 379/218 | |
| Peeters M-b | 2014 | Belgium | 486 | MT KRAS | Panitumumab+C | C | RCT | 1-180 | NR | |
| Heinemann V | 2014 | Germany | 592 | ITT | Bevacizumab+C | Cetuximab+C | RCT | 19-57 | 196/99 | 214/83 |
| Cao R | 2014 | China | 142 | ITT | Bevacizumab+C | C | RCT | NR | 40/25 | 48/29 |
| Ye LC | 2013 | China | 138 | WT KRAS | Cetuximab+C | C | RCT | median 25.0 | 46/24 | 42/26 |
| Tabernero J | 2013 | Spain | 198 | ITT | Sorafenib+C | C | RCT | median 22.4 | 42/55 | 63/38 |
| Siu LL | 2013 | USA | 750 | WT KRAS | Brivanib+Cetuximab+C | Cetuximab+C | RCT | 0.1-34.1 | 247/129 | 234/140 |
| Peeters M | 2013 | Spain | 144 | ITT | Trebananib+C | C | RCT | 3-30 | 60/35 | 24/25 |
| Infante JR-a | 2013 | USA | 85 | ITT | Axitinib+C | Bevacizumab+C | RCT | 0-54 | 25/17 | 28/15 |
| Infante JR-b | 2013 | USA | 83 | ITT | Axitinib+C | Axitinib+Bevacizumab+C | RCT | 0-54 | 25/17 | 26/15 |
| Infante JR-c | 2013 | USA | 84 | ITT | Bevacizumab+C | Axitinib+Bevacizumab+C | RCT | 0-54 | 28/15 | 26/15 |
| Cunningham D | 2013 | UK | 210 | ITT | Bevacizumab+C | Cediranib+C | RCT | 0-32 | 39/27 | 96/48 |
| Cohn AL-a | 2013 | USA | 103 | MT KRAS | Conatumumab+C | C | RCT | 0-22 | 27/24 | 23/29 |
| Cohn AL-b | 2013 | USA | 104 | MT KRAS | Ganitumab+C | C | RCT | 0-22 | 24/28 | 23/29 |
| Carrato A | 2013 | Singapore | 768 | ITT | Sunitinib+C | C | RCT | 0-30 | 222/164 | 203/179 |
| Bendell JC | 2013 | USA | 100 | ITT | Bevacizumab+C | Axitinib+C | RCT | 0-30 | 27/24 | 31/18 |
| Stintzing S | 2012 | Germany | 96 | ITT | Cetuximab+C | Bevacizumab+C | RCT | 0-48 | 32/18 | 30/16 |
| Schmoll HJ | 2012 | Germany | 1422 | ITT | Cediranib+C | Bevacizumab+C | RCT | median 14 | 412/297 | 414/299 |
| Saltz L | 2012 | USA | 247 | ITT | Bevacizumab+C | Cetuximab+Bevacizumab+C | RCT | 0-39 | 70/54 | 73/50 |
| Madajewicz S | 2012 | USA | 42 | ITT | Bevacizumab+C | C | RCT | NR | 11/7 | 9/15 |
| Matsusaka S | 2011 | Japan | 64 | ITT | Bevacizumab+C | C | RCT | NR | 15/18 | 16/15 |
| Guan ZZ | 2011 | China | 203 | ITT | Bevacizumab+C | C | RCT | 0-24 | 70/69 | 36/28 |
| Bokemeyer C | 2011 | Germany | 337 | ITT | Cetuximab+C | C | RCT | NR | 89/80 | 89/80 |
| Stathopoulos GP | 2010 | Greece | 222 | ITT | Bevacizumab+C | C | RCT | 36(12-72) | 73/41 | 68/40 |
| Van Cutsem E | 2009 | Belgium | 1198 | ITT | Cetuximab+C | C | RCT | median 29.7 | 369/230 | 356/243 |
| Santoro A | 2008 | Italy | 99 | ITT | Gefitinib+C | C | RCT | median 14.5 | 29/22 | 30/18 |
| Kohne CH | 2008 | Germany | 41 | ITT | Celecoxib+C | C | RCT | median 14.6 | 15/4 | 14/8 |
| Maiello E | 2006 | Italy | 81 | ITT | Cetuximab+C | C | RCT | 18(10-32) | 27/14 | 27/13 |

(Notes：RCT = randomized controlled trials; NR = not reported; M = male; F = female; C = chemotherapy; ITT = intention-to-treat; WT = wild-type; MT = mutant.)
