# Supplementary material for: A network meta-analysis on the efficacy of sixteen targeted drugs in combination with chemotherapy for treatment of advanced/metastatic colorectal cancer
Source: Oncotarget. 2016 Oct 31;7(51):84468–79. doi: 10.18632/oncotarget.12994 (PMC5356673; doi:10.18632/oncotarget.12994)
Supplement: Supplementary file 3 [file oncotarget-07-84468-s003.docx]

**Table S2:** Estimated OR and 95%CI produced by random effects pairwise meta-analysis for efficacy events in metastatic/advanced colorectal cancer patients.

| Outcomes | Included studies | Comparisons | Efficacy events | | Pairwise meta-analysis | |  |
| --- | --- | --- | --- | --- | --- | --- | --- |
|  |  |  | Treatment1 | Treatment2 | OR | 95% CI | |
| **SD** | 1 | B VS E | 53/95 | 44/96 | 1.217 | 0.746-1.987 | |
|  | 1 | B VS D | 50/91 | 35/91 | 1.429 | 0.849-2.404 | |
|  | 1 | B VS C | 85/295 | 53/297 | **2.517** | **1.639-3.866** | |
|  | 5 | B VS A | 171/369 | 133/304 | **0.556** | **0.433-0.715** | |
|  | 1 | B VS P | 13/43 | 11/41 | **0.110** | **0.054-0.224** | |
|  | 1 | B VS F | 38/66 | 80/144 | 1.564 | 0.941-2.601 | |
|  | 1 | B VS I | 15/51 | 20/49 | 0.497 | 0.238-1.038 | |
|  | 1 | B VS Q | 41/124 | 50/123 | **1.768** | **1.002-3.121** | |
|  | 4 | C VS A | 319/877 | 395/877 | **2.275** | **1.823-2.839** | |
|  | 1 | C VS B | 19/50 | 19/46 | 1.589 | 0.684-3.694 | |
|  | 2 | D VS A | 242/541 | 270/542 | 1.169 | 0.939-1.455 | |
|  | 1 | E VS A | 200/386 | 201/382 | **10.417** | **6.373-17.027** | |
|  | 1 | F VS B | 259/709 | 268/713 | 0.972 | 0.796-1.187 | |
|  | 1 | G VS A | 7/19 | 9/22 | **0.162** | **0.060-0.441** | |
|  | 1 | H VS A | 39/97 | 31/101 | **5.076** | **2.258-11.412** | |
|  | 1 | I VS B | 8/42 | 13/43 | 0.630 | 0.237-1.675 | |
|  | 1 | I VS P | 8/42 | 11/41 | **0.237** | **0.098-0.573** | |
|  | 1 | J VS A | 38/95 | 31/49 | **0.258** | **0.153-0.434** | |
|  | 1 | K VS A | 28/51 | 31/52 | 0.571 | 0.313-1.043 | |
|  | 1 | L VS A | 30/52 | 31/52 | **0.107** | **0.063-0.184** | |
|  | 1 | M VS A | 18/51 | 17/48 | 0.997 | 0.461-2.155 | |
|  | 1 | N VS A | 30/137 | 34/155 | **3.771** | **1.730-8.223** | |
|  | 1 | O VS C | 188/376 | 163/374 | **11.688** | **6.879-19.858** | |
| **PD** | 1 | B VS E | 1/95 | 3/96 | 0.337 | 0.034-3.296 | |
|  | 1 | B VS D | 11/91 | 12/91 | 0.917 | 0.385-2.184 | |
|  | 1 | B VS C | 16/295 | 21/297 | 0.767 | 0.392-1.499 | |
|  | 5 | B VS A | 34/369 | 63/304 | **0.533** | **0.346-0.823** | |
|  | 1 | B VS P | 5/43 | 3/41 | 1.589 | 0.357-7.079 | |
|  | 1 | B VS F | 9/66 | 31/144 | 0.633 | 0.285-1.406 | |
|  | 1 | B VS I | 11/51 | 15/49 | 0.705 | 0.295-1.684 | |
|  | 1 | B VS Q | 5/124 | 11/123 | 0.451 | 0.152-1.336 | |
|  | 4 | C VS A | 92/877 | 105/877 | 0.884 | 0.656-1.190 | |
|  | 1 | C VS B | 4/50 | 5/46 | 0.736 | 0.186-2.909 | |
|  | 2 | D VS A | 104/541 | 136/542 | 0.767 | 0.579-1.017 | |
|  | 1 | E VS A | 25/386 | 32/382 | 0.773 | 0.450-1.329 | |
|  | 1 | F VS B | 82/709 | 79/713 | 1.044 | 0.753-1.446 | |
|  | 1 | G VS A | 2/19 | 3/22 | 0.772 | 0.116-5.118 | |
|  | 1 | H VS A | 5/97 | 7/101 | 0.744 | 0.228-2.423 | |
|  | 1 | I VS B | 3/42 | 5/43 | 0.614 | 0.138-2.734 | |
|  | 1 | I VS P | 3/42 | 3/41 | 0.976 | 0.186-5.119 | |
|  | 1 | J VS A | 28/95 | 10/49 | 1.444 | 0.649-3.214 | |
|  | 1 | K VS A | 9/51 | 10/52 | 0.918 | 0.344-2.445 | |
|  | 1 | L VS A | 7/52 | 10/52 | 0.700 | 0.248-1.980 | |
|  | 1 | M VS A | 9/51 | 6/48 | 1.412 | 0.467-4.265 | |
|  | 1 | N VS A | 42/137 | 70/155 | 0.679 | 0.434-1.061 | |
|  | 1 | O VS C | 81/376 | 142/374 | **0.567** | **0.417-0.772** | |
| **CR** | 1 | B VS E | 1/95 | 1/96 | 1.011 | 0.062-16.391 | |
|  | 1 | B VS D | 1/91 | 1/91 | 1.000 | 0.062-16.232 | |
|  | 1 | B VS C | 4/295 | 13/297 | **0.310** | **0.100-0.961** | |
|  | 5 | B VS A | 14/369 | 9/304 | 1.476 | 0.614-3.546 | |
|  | 1 | B VS P | 2/43 | 1/41 | 1.907 | 0.166-21.841 | |
|  | 1 | B VS F | 1/66 | 1/144 | 2.182 | 0.134-35.419 | |
|  | 1 | B VS I | 1/51 | 1/49 | 0.961 | 0.058-15.790 | |
|  | 1 | B VS Q | 2/124 | 1/123 | 1.984 | 0.178-22.163 | |
|  | 4 | C VS A | 7/877 | 5/877 | 1.401 | 0.442-4.441 | |
|  | 1 | C VS B | 1/50 | 1/46 | 0.920 | 0.056-15.138 | |
|  | 2 | D VS A | 2/541 | 2/542 | 1.006 | 0.141-7.164 | |
|  | 1 | E VS A | 1/386 | 1/382 | 0.990 | 0.062-15.879 | |
|  | 1 | F VS B | 12/709 | 11/713 | 1.097 | 0.481-2.503 | |
|  | 1 | G VS A | 1/19 | 1/22 | 1.158 | 0.068-19.798 | |
|  | 1 | H VS A | 2/97 | 1/101 | 2.082 | 0.186-23.339 | |
|  | 1 | I VS B | 1/42 | 2/43 | 0.512 | 0.045-5.860 | |
|  | 1 | I VS P | 1/42 | 1/41 | 0.976 | 0.059-16.134 | |
|  | 1 | J VS A | 2/95 | 1/49 | 1.032 | 0.091-11.660 | |
|  | 1 | K VS A | 1/51 | 1/52 | 1.020 | 0.062-16.743 | |
|  | 1 | L VS A | 1/52 | 1/52 | 1.000 | 0.061-16.417 | |
|  | 1 | M VS A | 1/51 | 1/48 | 0.941 | 0.057-15.473 | |
|  | 1 | N VS A | 8/137 | 10/155 | 0.905 | 0.347-2.358 | |
|  | 1 | O VS C | 1/376 | 1S/374 | 0.995 | 0.062-15.961 | |
| **PR** | 1 | B VS E | 35/95 | 38/96 | 0.931 | 0.543-1.597 | |
|  | 1 | B VS D | 16/91 | 27/91 | 0.593 | 0.299-1.173 | |
|  | 1 | B VS C | 167/295 | 171/297 | 0.983 | 0.753-1.284 | |
|  | 5 | B VS A | 141/369 | 86/304 | **1.419** | **1.035-1.946** | |
|  | 1 | B VS P | 19/43 | 16/41 | 0.132 | 0.513-2.497 | |
|  | 1 | B VS F | 18/66 | 27/144 | 1.455 | 0.749-2.825 | |
|  | 1 | B VS I | 11/51 | 11/49 | 0.961 | 0.382-2.418 | |
|  | 1 | B VS Q | 59/124 | 48/123 | 1.219 | 0.773-1.922 | |
|  | 4 | C VS A | 408/877 | 322/877 | **1.267** | **1.065-1.507** | |
|  | 1 | C VS B | 17/50 | 21/46 | 0.745 | 0.350-1.584 | |
|  | 2 | D VS A | 137/541 | 63/542 | 2.119 | 1.536-2.923 | |
|  | 1 | E VS A | 124/386 | 127/382 | 0.966 | 0.727-1.285 | |
|  | 1 | F VS B | 316/709 | 326/713 | 0.975 | 0.809-1.175 | |
|  | 1 | G VS A | 5/19 | 10/22 | 0.579 | 0.168-1.994 | |
|  | 1 | H VS A | 43/97 | 60/101 | 0.746 | 0.461-1.207 | |
|  | 1 | I VS B | 11/42 | 19/43 | 0.593 | 0.252-1.395 | |
|  | 1 | I VS P | 11/42 | 16/41 | 0.671 | 0.278-1.618 | |
|  | 1 | J VS A | 10/95 | 1/49 | 5.158 | 0.642-41.465 | |
|  | 1 | K VS A | 7/51 | 1/52 | 7.137 | 0.848-60.095 | |
|  | 1 | L VS A | 4/52 | 1/52 | 4.000 | 0.432-37.005 | |
|  | 1 | M VS A | 22/51 | 22/48 | 0.941 | 0.463-1.915 | |
|  | 1 | N VS A | 47/137 | 37/155 | 1.437 | 0.882-2.342 | |
|  | 1 | O VS C | 51/376 | 27/374 | **1.879** | **1.153-3.061** | |
| **ORR** | 1 | B VS E | 36/95 | 39/96 | 0.933 | 0.547-1.592 | |
|  | 1 | B VS D | 17/91 | 28/91 | 0.607 | 0.311-1.185 | |
|  | 1 | B VS C | 171/295 | 184/297 | 0.936 | 0.719-1.217 | |
|  | 5 | B VS A | 155/369 | 95/304 | **1.420** | **1.048-1.925** | |
|  | 1 | B VS P | 21/43 | 17/41 | 1.178 | 0.546-2.542 | |
|  | 1 | B VS F | 19/66 | 28/144 | 1.481 | 0.772-2.840 | |
|  | 1 | B VS I | 12/51 | 12/49 | 0.961 | 0.394-2.342 | |
|  | 1 | B VS Q | 61/124 | 49/123 | 1.235 | 0.786-1.939 | |
|  | 4 | C VS A | 415/877 | 327/877 | **1.269** | **1.068-1.508** | |
|  | 1 | C VS B | 18/50 | 22/46 | 0.753 | 0.359-1.578 | |
|  | 2 | D VS A | 139/541 | 65/542 | **2.085** | **1.517-2.867** | |
|  | 1 | E VS A | 125/386 | 128/382 | 0.966 | 0.727-1.284 | |
|  | 1 | F VS B | 328/709 | 337/713 | 0.979 | 0.814-1.177 | |
|  | 1 | G VS A | 6/19 | 11/22 | 0.632 | 0.196-2.033 | |
|  | 1 | H VS A | 45/97 | 61/101 | 0.768 | 0.478-1.236 | |
|  | 1 | I VS B | 12/42 | 21/43 | 0.585 | 0.256-1.338 | |
|  | 1 | I VS P | 12/42 | 17/41 | 0.689 | 0.293-1.620 | |
|  | 1 | J VS A | 12/95 | 2/49 | 3.095 | 0.666-14.380 | |
|  | 1 | K VS A | 8/51 | 2/52 | 4.078 | 0.826-20.138 | |
|  | 1 | L VS A | 5/52 | 2/52 | 2.500 | 0.464-13.471 | |
|  | 1 | M VS A | 23/51 | 23/48 | 0.941 | 0.468-1.894 | |
|  | 1 | N VS A | 55/137 | 47/155 | 1.324 | 0.842-2.081 | |
|  | 1 | O VS C | 52/376 | 28/374 | **1.847** | **1.142-2.989** | |
| **DCR** | 1 | B VS E | 89/95 | 83/96 | 1.084 | 0.718-1.636 | |
|  | 1 | B VS D | 67/91 | 63/91 | 1.063 | 0.678-1.668 | |
|  | 1 | B VS C | 256/295 | 237/297 | 1.087 | 0.856-1.381 | |
|  | 5 | B VS A | 326/369 | 228/304 | 1.168 | 0.927-1.473 | |
|  | 1 | B VS P | 34/43 | 28/41 | 1.158 | 0.600-2.236 | |
|  | 1 | B VS F | 57/66 | 108/144 | 1.152 | 0.747-1.776 | |
|  | 1 | B VS I | 27/51 | 32/49 | 0.811 | 0.425-1.545 | |
|  | 1 | B VS Q | 102/124 | 99/123 | 1.022 | 0.704-1.483 | |
|  | 4 | C VS A | 734/877 | 722/877 | 1.018 | 0.886-1.169 | |
|  | 1 | C VS B | 37/50 | 41/46 | 0.830 | 0.456-1.510 | |
|  | 2 | D VS A | 381/541 | 335/542 | 1.138 | 0.942-1.375 | |
|  | 1 | E VS A | 325/386 | 329/382 | 0.978 | 0.794-1.204 | |
|  | 1 | F VS B | 587/709 | 605/713 | 0.976 | 0.837-1.138 | |
|  | 1 | G VS A | 13/19 | 20/22 | 0.753 | 0.297-1.907 | |
|  | 1 | H VS A | 84/97 | 92/101 | 0.951 | 0.633-1.427 | |
|  | 1 | I VS B | 20/42 | 34/43 | 0.602 | 0.300-1.209 | |
|  | 1 | I VS P | 20/42 | 28/41 | 0.697 | 0.340-1.429 | |
|  | 1 | J VS A | 50/95 | 33/49 | 0.781 | 0.447-1.366 | |
|  | 1 | K VS A | 36/51 | 33/52 | 1.112 | 0.604-2.047 | |
|  | 1 | L VS A | 35/52 | 33/52 | 1.061 | 0.575-1.955 | |
|  | 1 | M VS A | 41/51 | 40/48 | 0.965 | 0.536-1.736 | |
|  | 1 | N VS A | 85/137 | 81/155 | 1.187 | 0.811-1.738 | |
|  | 1 | O VS C | 240/376 | 191/374 | 1.250 | 0.985-1.586 | |

Notes: SD = stable disease; PD = progressive disease; CR = complete response; PR = partial response; ORR = overall response rate; DCR = disease control rate; ORR = CR+PR; DCR = SD+CR+PR; OR = odd ratios; 95% CI = 95% credible intervals; A: chemotherapy; B: bevacizumab+chemotherapy; C: cetuximab+chemotherapy; D: panitumumab+chemotherapy; E: sunitinib+chemotherapy; F: cediranib+chemotherapy; G: celecoxib+chemotherapy; H: sorafenib+chemotherapy; I = axitinib+chemotherapy; J = trebananib+chemotherapy; K = conatumumab+chemotherapy; L = ganitumab+chemotherapy; M = gefitinib+chemotherapy; N = panitumumab +bevacizumab+chemotherapy; O = brivanib+cetuximab+chemotherapy; P = axitinib+bevacizumab+chemotherapy; Q = cetuximab+bevacizumab+ chemotherapy.
