# Supplementary material for: A network meta-analysis on the efficacy of sixteen targeted drugs in combination with chemotherapy for treatment of advanced/metastatic colorectal cancer
Source: Oncotarget. 2016 Oct 31;7(51):84468–79. doi: 10.18632/oncotarget.12994 (PMC5356673; doi:10.18632/oncotarget.12994)
Supplement: Supplementary file 4 [file oncotarget-07-84468-s004.pdf]

Table S3. Odds ratios and 95% confidence intervals of 17 treatment modalities under six end indicators according to the network meta-analysis.

SD

Chemotherapy	1.12 (0.78, 1.54)	**0.69 (0.49, 0.98)**	0.77 (0.49, 1.20)	0.88 (0.49, 1.51)	1.05 (0.54, 1.94)	0.82 (0.20, 3.49)	1.52 (0.64, 3.65)	1.02 (0.45, 2.21)	**0.38 (0.15, 0.98)**	0.83 (0.29, 2.25)	0.93 (0.34, 2.52)	1.01 (0.35, 2.88)	0.98 (0.43, 2.32)	0.90 (0.42, 1.98)	1.18 (0.45, 3.04)	1.55 (0.63, 3.74)
0.90 (0.65, 1.28)	Bevacizumab+Chemotherapy	**0.62 (0.42, 0.94)**	0.69 (0.42, 1.17)	0.79 (0.44, 1.41)	0.94 (0.56, 1.60)	0.73 (0.17, 3.30)	1.37 (0.54, 3.53)	0.91 (0.45, 1.84)	**0.34 (0.13, 0.95)**	0.75 (0.25, 2.19)	0.83 (0.29, 2.37)	0.91 (0.30, 2.74)	0.89 (0.36, 2.21)	0.81 (0.38, 1.86)	1.06 (0.45, 2.55)	1.39 (0.61, 3.19)
1.44 (1.02, 2.05)	1.61 (1.06, 2.39)	Cetuximab+Chemotherapy	1.11 (0.63, 1.96)	1.27 (0.66, 2.38)	1.52 (0.77, 2.91)	1.18 (0.27, 5.34)	2.20 (0.87, 5.68)	1.47 (0.65, 3.31)	0.54 (0.20, 1.48)	1.20 (0.40, 3.45)	1.34 (0.46, 3.87)	1.46 (0.48, 4.37)	1.42 (0.58, 3.66)	1.29 (0.66, 2.64)	1.70 (0.64, 4.55)	2.23 (0.89, 5.48)
1.30 (0.83, 2.05)	1.45 (0.86, 2.40)	0.90 (0.51, 1.60)	Panitumumab+Chemotherapy	1.14 (0.57, 2.26)	1.36 (0.65, 2.82)	1.06 (0.24, 4.98)	1.97 (0.75, 5.32)	1.31 (0.55, 3.10)	0.49 (0.17, 1.40)	1.07 (0.35, 3.26)	1.20 (0.40, 3.59)	1.31 (0.41, 4.11)	1.28 (0.50, 3.40)	1.17 (0.48, 2.91)	1.54 (0.54, 4.27)	2.01 (0.76, 5.33)
1.14 (0.66, 2.03)	1.27 (0.71, 2.27)	0.79 (0.42, 1.51)	0.88 (0.44, 1.75)	Sunitinib+Chemotherapy	1.20 (0.54, 2.64)	0.94 (0.21, 4.50)	1.73 (0.64, 4.93)	1.16 (0.47, 2.84)	0.43 (0.15, 1.29)	0.94 (0.30, 2.98)	1.05 (0.34, 3.24)	1.15 (0.35, 3.85)	1.13 (0.42, 3.22)	1.03 (0.41, 2.71)	1.36 (0.46, 3.88)	1.76 (0.65, 4.84)
0.95 (0.52, 1.84)	1.06 (0.62, 1.79)	0.66 (0.34, 1.30)	0.73 (0.36, 1.54)	0.83 (0.38, 1.85)	Cediranib+Chemotherapy	0.79 (0.16, 3.79)	1.45 (0.50, 4.24)	0.98 (0.40, 2.28)	0.36 (0.12, 1.12)	0.79 (0.24, 2.62)	0.88 (0.28, 2.85)	0.96 (0.28, 3.26)	0.94 (0.34, 2.76)	0.86 (0.34, 2.32)	1.13 (0.41, 3.15)	1.47 (0.57, 3.95)
1.22 (0.29, 5.07)	1.36 (0.30, 5.76)	0.85 (0.19, 3.66)	0.94 (0.20, 4.10)	1.07 (0.22, 4.88)	1.27 (0.26, 6.07)	Celecoxib+Chemotherapy	1.85 (0.33, 10.11)	1.25 (0.23, 6.21)	0.46 (0.08, 2.52)	1.02 (0.18, 5.35)	1.12 (0.19, 6.10)	1.24 (0.20, 6.93)	1.21 (0.23, 6.18)	1.10 (0.21, 5.53)	1.44 (0.24, 7.90)	1.89 (0.34, 10.14)
0.66 (0.27, 1.57)	0.73 (0.28, 1.84)	0.46 (0.18, 1.15)	0.51 (0.19, 1.34)	0.58 (0.20, 1.56)	0.69 (0.24, 1.99)	0.54 (0.10, 2.99)	Sorafenib+Chemotherapy	0.67 (0.20, 2.10)	**0.25 (0.07, 0.89)**	0.54 (0.14, 2.09)	0.61 (0.16, 2.22)	0.66 (0.17, 2.59)	0.65 (0.19, 2.29)	0.60 (0.18, 1.93)	0.78 (0.21, 2.77)	1.01 (0.29, 3.56)
0.98 (0.45, 2.21)	1.09 (0.54, 2.20)	0.68 (0.30, 1.55)	0.76 (0.32, 1.81)	0.86 (0.35, 2.15)	1.02 (0.44, 2.52)	0.80 (0.16, 4.30)	1.50 (0.48, 5.04)	Axitinib+Chemotherapy	0.37 (0.11, 1.28)	0.81 (0.23, 3.04)	0.91 (0.26, 3.35)	0.99 (0.27, 3.64)	0.97 (0.32, 3.14)	0.88 (0.32, 2.65)	1.17 (0.47, 2.92)	1.51 (0.55, 4.64)
2.65 (1.02, 6.89)	2.95 (1.05, 7.90)	1.84 (0.67, 5.04)	2.05 (0.72, 5.85)	2.33 (0.77, 6.88)	2.79 (0.89, 8.68)	2.19 (0.40, 12.59)	4.06 (1.12, 14.48)	2.71 (0.78, 9.12)	Trebananib+Chemotherapy	2.20 (0.55, 8.75)	2.47 (0.65, 9.50)	2.67 (0.65, 11.15)	2.64 (0.75, 9.22)	2.39 (0.72, 8.20)	3.18 (0.82, 11.99)	**4.14 (1.11, 14.77)**
1.21 (0.44, 3.40)	1.34 (0.46, 3.99)	0.84 (0.29, 2.50)	0.93 (0.31, 2.83)	1.06 (0.34, 3.34)	1.27 (0.38, 4.17)	0.98 (0.19, 5.66)	1.85 (0.48, 6.96)	1.23 (0.33, 4.31)	0.46 (0.11, 1.83)	Conatumumab+Chemotherapy	1.12 (0.27, 4.60)	1.22 (0.27, 5.18)	1.19 (0.33, 4.38)	1.09 (0.31, 3.89)	1.43 (0.35, 5.77)	1.87 (0.49, 7.28)
1.08 (0.40, 2.94)	1.20 (0.42, 3.40)	0.74 (0.26, 2.17)	0.83 (0.28, 2.51)	0.95 (0.31, 2.94)	1.13 (0.35, 3.53)	0.89 (0.16, 5.25)	1.63 (0.45, 6.33)	1.10 (0.30, 3.91)	0.41 (0.11, 1.54)	0.89 (0.22, 3.68)	Ganitumab+Chemotherapy	1.09 (0.26, 4.58)	1.06 (0.29, 4.00)	0.97 (0.27, 3.55)	1.28 (0.32, 5.00)	1.66 (0.44, 6.52)
0.99 (0.35, 2.85)	1.10 (0.36, 3.33)	0.68 (0.23, 2.10)	0.76 (0.24, 2.44)	0.87 (0.26, 2.86)	1.04 (0.31, 3.51)	0.81 (0.14, 4.89)	1.51 (0.39, 5.90)	1.01 (0.27, 3.72)	0.37 (0.09, 1.54)	0.82 (0.19, 3.64)	0.92 (0.22, 3.85)	Gefitinib+Chemotherapy	0.98 (0.26, 3.82)	0.89 (0.25, 3.44)	1.18 (0.29, 4.74)	1.53 (0.40, 5.92)
1.02 (0.43, 2.34)	1.13 (0.45, 2.76)	0.70 (0.27, 1.73)	0.78 (0.29, 1.98)	0.89 (0.31, 2.40)	1.06 (0.36, 2.97)	0.82 (0.16, 4.34)	1.55 (0.45, 5.13)	1.04 (0.32, 3.16)	0.38 (0.11, 1.34)	0.84 (0.23, 3.05)	0.94 (0.25, 3.44)	1.02 (0.26, 3.80)	PB+Chemotherapy	0.91 (0.28, 2.87)	1.21 (0.33, 4.13)	1.57 (0.47, 5.19)
1.11 (0.51, 2.40)	1.24 (0.54, 2.66)	0.77 (0.38, 1.53)	0.86 (0.34, 2.08)	0.98 (0.37, 2.44)	1.17 (0.43, 2.90)	0.91 (0.18, 4.71)	1.68 (0.52, 5.41)	1.13 (0.38, 3.17)	0.42 (0.12, 1.40)	0.92 (0.26, 3.23)	1.03 (0.28, 3.67)	1.13 (0.29, 4.07)	1.10 (0.35, 3.59)	BC+Chemotherapy	1.31 (0.38, 4.28)	1.72 (0.56, 5.27)
0.85 (0.33, 2.20)	0.94 (0.39, 2.23)	0.59 (0.22, 1.56)	0.65 (0.23, 1.84)	0.74 (0.26, 2.17)	0.89 (0.32, 2.42)	0.69 (0.13, 4.11)	1.28 (0.36, 4.72)	0.86 (0.34, 2.13)	0.31 (0.08, 1.22)	0.70 (0.17, 2.86)	0.78 (0.20, 3.08)	0.85 (0.21, 3.49)	0.83 (0.24, 3.03)	0.77 (0.23, 2.65)	AB+Chemotherapy	1.31 (0.40, 4.54)
0.65 (0.27, 1.58)	0.72 (0.31, 1.63)	0.45 (0.18, 1.13)	0.50 (0.19, 1.30)	0.57 (0.21, 1.55)	0.68 (0.25, 1.77)	0.53 (0.10, 2.91)	0.99 (0.28, 3.41)	0.66 (0.22, 1.83)	0.24 (0.07, 1.00)	0.53 (0.14, 2.02)	0.60 (0.15, 2.50)	0.65 (0.17, 2.50)	0.64 (0.19, 2.13)	0.58 (0.19, 1.80)	0.76 (0.22, 2.49)	CB+Chemotherapy

PD

Chemotherapy	**0.51 (0.29, 0.77)**	0.77 (0.44, 1.13)	0.69 (0.37, 1.15)	0.82 (0.36, 2.04)	0.63 (0.27, 1.39)	0.57 (0.06, 5.30)	0.72 (0.16, 3.10)	0.54 (0.17, 1.43)	1.69 (0.56, 5.38)	0.93 (0.26, 3.27)	0.56 (0.16, 2.40)	1.54 (0.39, 6.86)	0.53 (0.21, 1.36)	**0.34 (0.12, 0.84)**	0.37 (0.09, 1.50)	1.21 (0.29, 5.07)
1.96 (1.30, 3.48)	Bevacizumab+Chemotherapy	1.52 (0.87, 2.68)	1.38 (0.72, 2.73)	1.62 (0.69, 4.79)	1.22 (0.66, 2.61)	1.16 (0.11, 11.35)	1.44 (0.30, 6.98)	1.07 (0.42, 2.57)	**3.37 (1.01, 12.15)**	1.89 (0.47, 7.35)	1.12 (0.32, 5.75)	3.09 (0.78, 15.21)	1.05 (0.41, 3.30)	0.66 (0.24, 1.92)	0.72 (0.18, 2.81)	2.37 (0.66, 9.63)
1.29 (0.88, 2.27)	0.66 (0.37, 1.15)	Cetuximab+Chemotherapy	0.89 (0.45, 1.89)	1.08 (0.45, 3.22)	0.81 (0.36, 2.10)	0.77 (0.07, 7.26)	0.94 (0.21, 4.73)	0.71 (0.23, 2.01)	2.21 (0.61, 7.92)	1.23 (0.33, 4.80)	0.75 (0.21, 3.42)	1.99 (0.50, 11.40)	0.69 (0.27, 2.15)	0.44 (0.19, 1.09)	0.49 (0.11, 2.14)	1.59 (0.39, 7.36)
1.44 (0.87, 2.69)	0.72 (0.37, 1.40)	1.12 (0.53, 2.24)	Panitumumab+Chemotherapy	1.20 (0.46, 3.65)	0.89 (0.36, 2.44)	0.86 (0.08, 8.04)	1.07 (0.22, 5.09)	0.78 (0.24, 2.31)	2.45 (0.70, 8.93)	1.37 (0.34, 5.40)	0.83 (0.21, 3.92)	2.28 (0.53, 11.25)	0.77 (0.27, 2.43)	0.49 (0.16, 1.51)	0.53 (0.12, 2.38)	1.75 (0.42, 7.90)
1.22 (0.49, 2.80)	0.62 (0.21, 1.46)	0.93 (0.31, 2.25)	0.83 (0.27, 2.15)	Sunitinib+Chemotherapy	0.77 (0.22, 2.30)	0.72 (0.06, 6.48)	0.92 (0.15, 4.57)	0.65 (0.15, 2.25)	1.95 (0.50, 8.33)	1.14 (0.23, 4.94)	0.67 (0.15, 4.29)	1.99 (0.35, 10.06)	0.65 (0.17, 2.27)	0.42 (0.10, 1.37)	0.42 (0.08, 2.18)	1.45 (0.27, 8.98)
1.58 (0.72, 3.74)	0.82 (0.38, 1.51)	1.23 (0.48, 2.80)	1.12 (0.41, 2.81)	1.30 (0.43, 4.51)	Cediranib+Chemotherapy	0.92 (0.09, 9.59)	1.14 (0.21, 6.26)	0.86 (0.26, 2.55)	2.71 (0.70, 11.05)	1.51 (0.33, 6.86)	0.90 (0.21, 4.97)	2.44 (0.52, 14.04)	0.85 (0.26, 3.09)	0.54 (0.16, 1.80)	0.59 (0.12, 2.64)	1.93 (0.44, 9.04)
1.76 (0.19, 16.90)	0.86 (0.09, 9.04)	1.30 (0.14, 13.64)	1.17 (0.12, 11.48)	1.40 (0.15, 16.23)	1.09 (0.10, 11.72)	Celecoxib+Chemotherapy	1.33 (0.08, 17.92)	0.90 (0.07, 9.92)	2.63 (0.26, 35.95)	1.59 (0.13, 19.68)	1.00 (0.06, 14.11)	2.70 (0.21, 38.98)	0.92 (0.09, 10.91)	0.58 (0.05, 6.88)	0.57 (0.05, 8.44)	1.96 (0.15, 26.92)
1.39 (0.32, 6.38)	0.69 (0.14, 3.33)	1.06 (0.21, 4.85)	0.94 (0.20, 4.59)	1.09 (0.22, 6.51)	0.88 (0.16, 4.81)	0.75 (0.06, 11.92)	Sorafenib+Chemotherapy	0.72 (0.11, 4.48)	2.15 (0.37, 15.67)	1.23 (0.19, 9.16)	0.74 (0.12, 6.55)	2.09 (0.28, 17.04)	0.72 (0.13, 4.30)	0.47 (0.08, 2.63)	0.48 (0.07, 4.38)	1.71 (0.22, 12.51)
1.85 (0.70, 5.83)	0.93 (0.39, 2.37)	1.40 (0.50, 4.27)	1.28 (0.43, 4.22)	1.54 (0.44, 6.84)	1.16 (0.39, 3.88)	1.11 (0.10, 13.40)	1.39 (0.22, 8.79)	Axitinib+Chemotherapy	3.16 (0.64, 15.90)	1.72 (0.36, 9.54)	1.08 (0.22, 6.86)	2.89 (0.59, 19.66)	1.00 (0.27, 4.51)	0.61 (0.17, 2.60)	0.69 (0.12, 2.78)	2.29 (0.47, 11.80)
0.59 (0.19, 1.78)	0.30 (0.08, 1.00)	0.45 (0.13, 1.63)	0.41 (0.11, 1.43)	0.51 (0.12, 2.01)	0.37 (0.09, 1.42)	0.38 (0.03, 3.84)	0.47 (0.06, 2.70)	0.32 (0.06, 1.56)	Trebananib+Chemotherapy	0.55 (0.10, 2.96)	0.36 (0.06, 1.93)	0.99 (0.16, 5.59)	0.32 (0.07, 1.36)	**0.20 (0.04, 0.80)**	0.21 (0.03, 1.27)	0.70 (0.11, 7.22)
1.07 (0.31, 3.88)	0.53 (0.14, 2.11)	0.81 (0.21, 3.00)	0.73 (0.19, 2.91)	0.88 (0.20, 4.35)	0.66 (0.15, 3.01)	0.63 (0.05, 7.63)	0.81 (0.11, 5.24)	0.58 (0.10, 2.80)	1.82 (0.34, 9.70)	Conatumumab+Chemotherapy	0.61 (0.10, 4.50)	1.62 (0.26, 11.61)	0.57 (0.13, 2.75)	0.37 (0.07, 1.70)	0.41 (0.06, 2.74)	1.40 (0.18, 9.15)
1.78 (0.42, 6.10)	0.89 (0.17, 3.15)	1.34 (0.29, 4.78)	1.20 (0.26, 4.68)	1.49 (0.23, 6.67)	1.12 (0.20, 4.69)	1.00 (0.07, 17.16)	1.35 (0.15, 8.02)	0.93 (0.15, 4.60)	2.79 (0.52, 15.64)	1.65 (0.22, 9.96)	Ganitumab+Chemotherapy	2.80 (0.39, 18.91)	0.93 (0.18, 4.67)	0.60 (0.10, 2.71)	0.58 (0.09, 4.27)	2.17 (0.27, 16.19)
0.65 (0.15, 2.59)	0.32 (0.07, 1.29)	0.50 (0.10, 2.00)	0.44 (0.09, 1.90)	0.50 (0.10, 2.87)	0.41 (0.07, 1.91)	0.37 (0.03, 4.75)	0.48 (0.06, 3.53)	0.35 (0.05, 1.70)	1.01 (0.18, 6.41)	0.62 (0.09, 3.86)	0.36 (0.05, 2.57)	Gefitinib+Chemotherapy	0.34 (0.06, 1.85)	0.22 (0.03, 1.14)	0.22 (0.03, 1.74)	0.77 (0.10, 5.48)
1.89 (0.73, 4.68)	0.95 (0.30, 2.46)	1.45 (0.47, 3.70)	1.31 (0.41, 3.71)	1.54 (0.44, 5.72)	1.18 (0.32, 3.83)	1.08 (0.09, 10.92)	1.39 (0.23, 9.42)	1.00 (0.22, 3.72)	3.09 (0.74, 14.28)	1.75 (0.36, 7.96)	1.07 (0.22, 5.53)	2.98 (0.54, 16.20)	PB+Chemotherapy	0.65 (0.15, 2.26)	0.66 (0.12, 3.67)	2.21 (0.40, 13.43)
2.93 (1.19, 8.38)	1.52 (0.52, 4.15)	2.29 (0.92, 5.29)	2.06 (0.66, 6.21)	2.40 (0.73, 10.19)	1.85 (0.56, 6.43)	1.71 (0.15, 19.61)	2.15 (0.38, 12.88)	1.65 (0.38, 5.73)	4.92 (1.25, 23.11)	2.70 (0.59, 14.34)	1.67 (0.37, 9.84)	4.46 (0.88, 28.73)	1.54 (0.44, 6.61)	BC+Chemotherapy	1.09 (0.21, 5.88)	3.62 (0.68, 19.77)
2.73 (0.67, 11.40)	1.38 (0.36, 5.41)	2.04 (0.47, 9.12)	1.90 (0.42, 8.22)	2.38 (0.46, 12.60)	1.70 (0.38, 8.14)	1.76 (0.12, 21.24)	2.07 (0.23, 15.09)	1.45 (0.36, 8.46)	4.72 (0.78, 28.89)	2.45 (0.37, 16.88)	1.73 (0.23, 11.72)	4.54 (0.57, 34.95)	1.52 (0.27, 8.47)	0.92 (0.17, 4.80)	AB+Chemotherapy	3.31 (0.47, 40.31)
0.83 (0.20, 3.41)	0.42 (0.10, 1.52)	0.63 (0.14, 2.57)	0.57 (0.13, 2.41)	0.69 (0.11, 3.74)	0.52 (0.11, 2.29)	0.51 (0.04, 6.71)	0.59 (0.08, 4.46)	0.44 (0.08, 2.11)	1.44 (0.14, 8.84)	0.71 (0.11, 5.45)	0.46 (0.06, 3.65)	1.30 (0.18, 10.01)	0.45 (0.07, 2.49)	0.28 (0.05, 1.46)	0.30 (0.02, 2.11)	CB+Chemotherapy

CR

Chemotherapy	1.18 (0.51, 2.77)	2.12 (0.81, 7.38)	1.17 (0.19, 6.52)	1.13 (0.09, 14.43)	1.17 (0.27, 4.91)	1.10 (0.02, 41.02)	2.00 (0.18, 51.48)	0.68 (0.06, 4.71)	1.07 (0.06, 33.57)	0.95 (0.02, 42.92)	1.01 (0.04, 25.36)	1.19 (0.05, 30.94)	0.95 (0.24, 3.33)	2.33 (0.05, 168.30)	0.56 (0.03, 6.20)	0.36 (0.01, 6.57)
0.85 (0.36, 1.95)	Bevacizumab+Chemotherapy	1.80 (0.67, 6.09)	1.02 (0.13, 5.85)	0.93 (0.09, 11.43)	0.95 (0.32, 2.98)	0.88 (0.02, 41.28)	1.71 (0.14, 50.09)	0.53 (0.08, 3.16)	0.85 (0.04, 31.97)	0.82 (0.02, 43.03)	0.88 (0.03, 24.88)	1.01 (0.04, 28.04)	0.82 (0.15, 4.25)	1.93 (0.03, 146.37)	0.41 (0.03, 4.65)	0.30 (0.01, 4.53)
0.47 (0.14, 1.23)	0.56 (0.16, 1.50)	Cetuximab+Chemotherapy	0.59 (0.06, 3.45)	0.46 (0.03, 8.42)	0.54 (0.12, 2.37)	0.49 (0.01, 19.94)	0.92 (0.07, 29.54)	0.32 (0.04, 2.26)	0.50 (0.02, 14.60)	0.41 (0.01, 23.67)	0.48 (0.02, 13.81)	0.54 (0.02, 17.47)	0.45 (0.08, 2.23)	1.12 (0.02, 66.37)	0.25 (0.02, 3.22)	0.17 (0.00, 3.26)
0.85 (0.15, 5.19)	0.98 (0.17, 7.86)	1.70 (0.29, 15.81)	Panitumumab+Chemotherapy	1.02 (0.06, 18.82)	0.94 (0.12, 10.60)	0.93 (0.02, 46.98)	1.82 (0.10, 88.88)	0.57 (0.04, 8.18)	0.94 (0.03, 52.00)	0.86 (0.01, 58.58)	0.97 (0.02, 32.25)	1.05 (0.03, 35.49)	0.82 (0.08, 7.07)	1.86 (0.03, 235.85)	0.42 (0.02, 7.39)	0.27 (0.01, 8.67)
0.88 (0.07, 10.54)	1.08 (0.09, 11.61)	2.16 (0.12, 28.67)	0.98 (0.05, 16.48)	Sunitinib+Chemotherapy	1.07 (0.06, 13.84)	0.99 (0.01, 72.02)	2.28 (0.04, 110.16)	0.59 (0.02, 16.22)	0.89 (0.01, 70.01)	0.78 (0.01, 90.31)	0.78 (0.02, 51.00)	1.05 (0.02, 46.68)	0.87 (0.04, 12.63)	2.29 (0.02, 220.73)	0.40 (0.02, 17.28)	0.31 (0.00, 11.96)
0.85 (0.20, 3.65)	1.05 (0.34, 3.08)	1.84 (0.42, 8.69)	1.07 (0.09, 8.52)	0.94 (0.07, 15.46)	Cediranib+Chemotherapy	0.89 (0.02, 45.18)	1.79 (0.11, 70.33)	0.54 (0.07, 4.41)	0.90 (0.03, 40.87)	0.83 (0.01, 51.02)	0.93 (0.02, 29.25)	1.05 (0.03, 35.05)	0.85 (0.10, 5.16)	2.02 (0.03, 134.84)	0.44 (0.02, 6.76)	0.31 (0.01, 5.37)
0.91 (0.02, 44.60)	1.13 (0.02, 56.24)	2.03 (0.05, 92.42)	1.08 (0.02, 55.55)	1.02 (0.01, 130.38)	1.13 (0.02, 65.54)	Celecoxib+Chemotherapy	1.81 (0.03, 297.10)	0.60 (0.01, 37.66)	1.16 (0.01, 194.93)	0.94 (0.00, 177.49)	0.95 (0.01, 222.82)	1.13 (0.01, 176.74)	0.95 (0.02, 54.18)	2.34 (0.01, 916.66)	0.53 (0.00, 47.82)	0.30 (0.00, 42.89)
0.50 (0.02, 5.48)	0.58 (0.02, 7.20)	1.09 (0.03, 13.84)	0.55 (0.01, 10.17)	0.44 (0.01, 25.18)	0.56 (0.01, 8.99)	0.55 (0.00, 32.42)	Sorafenib+Chemotherapy	0.36 (0.00, 6.88)	0.45 (0.00, 31.70)	0.36 (0.00, 45.12)	0.43 (0.01, 21.31)	0.52 (0.01, 34.60)	0.45 (0.01, 7.26)	1.22 (0.01, 159.43)	0.26 (0.01, 5.46)	0.15 (0.00, 7.50)
1.47 (0.21, 17.08)	1.87 (0.32, 12.64)	3.10 (0.44, 25.24)	1.75 (0.12, 23.39)	1.70 (0.06, 41.89)	1.86 (0.23, 14.51)	1.68 (0.03, 92.22)	2.76 (0.15, 223.27)	Axitinib+Chemotherapy	1.74 (0.05, 69.86)	1.29 (0.03, 110.31)	1.66 (0.03, 67.55)	1.93 (0.04, 91.48)	1.46 (0.13, 25.81)	3.47 (0.03, 419.88)	0.82 (0.06, 7.83)	0.51 (0.01, 13.97)
0.94 (0.03, 17.28)	1.17 (0.03, 24.54)	2.01 (0.07, 46.40)	1.06 (0.02, 31.09)	1.12 (0.01, 83.11)	1.11 (0.02, 38.54)	0.86 (0.01, 167.61)	2.21 (0.03, 205.12)	0.57 (0.01, 21.86)	Trebananib+Chemotherapy	0.74 (0.01, 57.38)	0.87 (0.01, 124.81)	1.32 (0.01, 77.31)	0.98 (0.02, 22.25)	1.99 (0.02, 405.93)	0.48 (0.00, 20.46)	0.32 (0.00, 23.67)
1.06 (0.02, 45.15)	1.22 (0.02, 61.77)	2.41 (0.04, 138.68)	1.16 (0.02, 78.69)	1.29 (0.01, 157.72)	1.20 (0.02, 75.03)	1.06 (0.01, 249.22)	2.77 (0.02, 308.33)	0.78 (0.01, 36.35)	1.35 (0.02, 143.65)	Conatumumab+Chemotherapy	1.02 (0.01, 136.94)	1.50 (0.01, 144.11)	1.00 (0.02, 56.02)	2.94 (0.01, 660.72)	0.66 (0.00, 43.29)	0.39 (0.00, 55.16)
0.99 (0.04, 23.12)	1.13 (0.04, 34.13)	2.07 (0.07, 63.53)	1.04 (0.03, 44.14)	1.29 (0.02, 62.36)	1.08 (0.03, 41.54)	1.06 (0.00, 100.47)	2.31 (0.05, 141.06)	0.60 (0.01, 35.24)	1.16 (0.01, 85.55)	0.98 (0.01, 99.65)	Ganitumab+Chemotherapy	1.03 (0.01, 103.19)	0.93 (0.03, 22.76)	2.32 (0.02, 297.90)	0.46 (0.01, 35.07)	0.30 (0.01, 29.09)
0.84 (0.03, 21.43)	0.99 (0.04, 26.76)	1.86 (0.06, 52.58)	0.95 (0.03, 32.96)	0.95 (0.02, 56.74)	0.95 (0.03, 29.40)	0.88 (0.01, 100.82)	1.91 (0.03, 146.68)	0.52 (0.01, 23.36)	0.76 (0.01, 142.03)	0.67 (0.01, 114.89)	0.97 (0.01, 75.23)	Gefitinib+Chemotherapy	0.75 (0.02, 24.21)	2.14 (0.01, 336.04)	0.42 (0.01, 19.96)	0.29 (0.00, 23.43)
1.06 (0.30, 4.22)	1.22 (0.25, 6.65)	2.21 (0.45, 13.19)	1.22 (0.14, 12.92)	1.15 (0.08, 22.56)	1.18 (0.19, 9.79)	1.05 (0.02, 62.14)	2.24 (0.14, 77.77)	0.69 (0.04, 7.77)	1.02 (0.04, 43.67)	1.00 (0.02, 54.68)	1.08 (0.04, 39.63)	1.34 (0.04, 41.78)	PB+Chemotherapy	2.24 (0.04, 187.18)	0.52 (0.03, 9.56)	0.33 (0.01, 8.79)
0.43 (0.01, 20.55)	0.52 (0.01, 31.02)	0.89 (0.02, 50.56)	0.54 (0.00, 37.92)	0.44 (0.00, 46.14)	0.50 (0.01, 37.40)	0.43 (0.00, 76.29)	0.82 (0.01, 160.53)	0.29 (0.00, 31.75)	0.50 (0.00, 61.72)	0.34 (0.00, 108.15)	0.43 (0.00, 48.67)	0.47 (0.00, 115.74)	0.45 (0.01, 23.65)	BC+Chemotherapy	0.20 (0.00, 26.15)	0.12 (0.00, 29.74)
1.79 (0.16, 32.83)	2.42 (0.22, 32.86)	3.96 (0.31, 62.07)	2.40 (0.14, 47.52)	2.52 (0.06, 51.66)	2.29 (0.15, 46.16)	1.88 (0.02, 209.15)	3.91 (0.18, 195.29)	1.22 (0.13, 16.18)	2.06 (0.05, 243.98)	1.51 (0.02, 217.83)	2.15 (0.03, 119.85)	2.40 (0.05, 127.80)	1.93 (0.10, 30.83)	4.96 (0.04, 422.46)	AB+Chemotherapy	0.65 (0.02, 23.84)
2.77 (0.15, 93.97)	3.32 (0.22, 134.44)	6.04 (0.31, 267.90)	3.77 (0.12, 108.70)	3.18 (0.08, 213.96)	3.26 (0.19, 144.32)	3.38 (0.02, 282.52)	6.71 (0.13, 495.13)	1.96 (0.07, 87.45)	3.10 (0.04, 335.89)	2.55 (0.02, 353.63)	3.37 (0.03, 193.08)	3.44 (0.04, 388.53)	3.00 (0.11, 109.31)	8.11 (0.03, 1158.92)	1.55 (0.04, 60.26)	CB+Chemotherapy

PR

Chemotherapy	**1.65 (1.07, 2.67)**	1.60 (0.98, 2.64)	**2.48 (1.27, 4.80)**	1.26 (0.58, 2.82)	1.33 (0.56, 3.30)	0.42 (0.07, 2.19)	0.54 (0.17, 1.75)	1.02 (0.39, 2.76)	8.12 (0.89, 222.55)	**10.84 (1.19, 407.09)**	5.37 (0.55, 203.85)	0.89 (0.24, 3.01)	1.66 (0.56, 5.13)	3.23 (0.95, 11.03)	1.54 (0.52, 4.87)	1.15 (0.36, 3.89)
0.61 (0.38, 0.94)	Bevacizumab+Chemotherapy	0.97 (0.55, 1.65)	1.50 (0.70, 3.05)	0.77 (0.34, 1.70)	0.80 (0.37, 1.75)	0.25 (0.04, 1.40)	0.33 (0.09, 1.12)	0.62 (0.26, 1.45)	4.96 (0.52, 135.16)	6.54 (0.68, 261.89)	3.22 (0.32, 121.02)	0.54 (0.14, 1.96)	1.01 (0.31, 3.32)	1.96 (0.55, 6.85)	0.93 (0.34, 2.66)	0.70 (0.23, 2.09)
0.63 (0.38, 1.02)	1.03 (0.61, 1.83)	Cetuximab+Chemotherapy	1.56 (0.68, 3.38)	0.79 (0.33, 1.98)	0.83 (0.33, 2.14)	0.26 (0.04, 1.44)	0.34 (0.10, 1.20)	0.63 (0.24, 1.79)	5.15 (0.53, 143.78)	6.74 (0.70, 268.73)	3.38 (0.32, 124.79)	0.56 (0.14, 2.10)	1.04 (0.31, 3.59)	2.03 (0.66, 6.06)	0.96 (0.31, 3.13)	0.72 (0.22, 2.46)
0.40 (0.21, 0.79)	0.67 (0.33, 1.42)	0.64 (0.30, 1.47)	Panitumumab+Chemotherapy	0.51 (0.19, 1.41)	0.53 (0.19, 1.60)	**0.17 (0.03, 0.98)**	**0.22 (0.06, 0.86)**	0.41 (0.14, 1.32)	3.27 (0.33, 89.18)	4.42 (0.44, 181.42)	2.16 (0.20, 87.75)	0.36 (0.08, 1.49)	0.67 (0.19, 2.49)	1.31 (0.33, 5.32)	0.62 (0.18, 2.25)	0.47 (0.12, 1.75)
0.79 (0.35, 1.72)	1.30 (0.59, 2.90)	1.27 (0.51, 3.03)	1.97 (0.71, 5.21)	Sunitinib+Chemotherapy	1.06 (0.34, 3.16)	0.33 (0.05, 2.07)	0.43 (0.10, 1.71)	0.81 (0.24, 2.60)	6.55 (0.62, 186.85)	8.67 (0.82, 349.49)	4.21 (0.37, 177.77)	0.70 (0.16, 3.00)	1.32 (0.34, 5.04)	2.58 (0.59, 10.44)	1.22 (0.33, 4.40)	0.91 (0.23, 3.55)
0.75 (0.30, 1.80)	1.24 (0.57, 2.71)	1.20 (0.47, 3.06)	1.87 (0.63, 5.39)	0.95 (0.32, 2.92)	Cediranib+Chemotherapy	0.31 (0.05, 2.04)	0.41 (0.09, 1.79)	0.76 (0.24, 2.47)	6.20 (0.56, 182.87)	8.25 (0.76, 324.01)	4.06 (0.34, 159.22)	0.66 (0.14, 3.05)	1.25 (0.30, 5.30)	2.44 (0.58, 10.29)	1.16 (0.34, 4.19)	0.86 (0.23, 3.39)
2.37 (0.46, 13.44)	3.95 (0.72, 23.50)	3.83 (0.70, 22.95)	5.99 (1.02, 37.26)	2.99 (0.48, 20.56)	3.18 (0.49, 21.79)	Celecoxib+Chemotherapy	1.30 (0.18, 10.16)	2.45 (0.35, 17.27)	**20.52 (1.26, 774.58)**	**26.47 (1.69, 1619.40)**	13.39 (0.77, 802.51)	2.10 (0.27, 18.13)	3.91 (0.56, 31.34)	**7.82 (1.01, 61.58)**	3.70 (0.52, 28.18)	2.74 (0.37, 22.38)
1.84 (0.57, 5.86)	3.02 (0.89, 10.83)	2.93 (0.83, 10.15)	4.54 (1.16, 17.17)	2.30 (0.58, 9.56)	2.44 (0.56, 10.70)	0.77 (0.10, 5.52)	Sorafenib+Chemotherapy	1.87 (0.42, 8.81)	**14.94 (1.26, 477.66)**	**20.27 (1.64, 948.91)**	10.30 (0.75, 420.53)	1.64 (0.30, 8.64)	3.04 (0.63, 15.04)	**5.96 (1.14, 31.59)**	2.83 (0.57, 14.82)	2.10 (0.40, 11.54)
0.98 (0.36, 2.57)	1.62 (0.69, 3.83)	1.58 (0.56, 4.21)	2.44 (0.76, 7.31)	1.23 (0.39, 4.09)	1.31 (0.40, 4.17)	0.41 (0.06, 2.82)	0.54 (0.11, 2.36)	Axitinib+Chemotherapy	8.09 (0.70, 255.85)	10.81 (0.91, 487.86)	5.47 (0.43, 220.40)	0.88 (0.17, 4.29)	1.65 (0.36, 6.89)	3.20 (0.69, 14.48)	1.51 (0.54, 4.30)	1.13 (0.28, 4.45)
0.12 (0.00, 1.12)	0.20 (0.01, 1.94)	0.19 (0.01, 1.89)	0.31 (0.01, 3.05)	0.15 (0.01, 1.60)	0.16 (0.01, 1.78)	0.05 (0.00, 0.79)	0.07 (0.00, 0.79)	0.12 (0.00, 1.43)	Trebananib+Chemotherapy	1.34 (0.02, 93.97)	0.67 (0.01, 41.08)	0.11 (0.00, 1.42)	0.20 (0.01, 2.38)	0.40 (0.01, 5.07)	0.19 (0.01, 2.30)	0.14 (0.00, 1.82)
0.09 (0.00, 0.84)	0.15 (0.00, 1.47)	0.15 (0.00, 1.42)	0.23 (0.01, 2.26)	0.12 (0.00, 1.22)	0.12 (0.00, 1.32)	0.04 (0.00, 0.59)	0.05 (0.00, 0.61)	0.09 (0.00, 1.09)	0.74 (0.01, 40.83)	Conatumumab+Chemotherapy	0.50 (0.01, 40.73)	0.08 (0.00, 1.04)	0.15 (0.00, 1.79)	0.29 (0.01, 3.78)	0.14 (0.00, 1.78)	0.10 (0.00, 1.29)
0.19 (0.00, 1.82)	0.31 (0.01, 3.17)	0.30 (0.01, 3.10)	0.46 (0.01, 4.99)	0.24 (0.01, 2.68)	0.25 (0.01, 2.95)	0.07 (0.00, 1.29)	0.10 (0.00, 1.34)	0.18 (0.00, 2.32)	1.49 (0.02, 76.44)	1.99 (0.02, 156.63)	Ganitumab+Chemotherapy	0.16 (0.00, 2.26)	0.30 (0.01, 4.41)	0.60 (0.01, 8.13)	0.28 (0.01, 3.75)	0.21 (0.01, 2.83)
1.12 (0.33, 4.09)	1.86 (0.51, 7.20)	1.79 (0.48, 6.98)	2.78 (0.67, 11.96)	1.42 (0.33, 6.40)	1.50 (0.33, 6.92)	0.48 (0.06, 3.73)	0.61 (0.12, 3.36)	1.14 (0.23, 5.90)	9.41 (0.70, 317.50)	12.44 (0.96, 587.44)	6.34 (0.44, 282.57)	Gefitinib+Chemotherapy	1.87 (0.35, 10.22)	3.60 (0.69, 21.56)	1.74 (0.34, 9.40)	1.30 (0.23, 7.56)
0.60 (0.19, 1.78)	0.99 (0.30, 3.25)	0.97 (0.28, 3.20)	1.49 (0.40, 5.30)	0.76 (0.20, 2.95)	0.80 (0.19, 3.35)	0.26 (0.03, 1.78)	0.33 (0.07, 1.58)	0.61 (0.15, 2.79)	4.97 (0.42, 164.31)	6.67 (0.56, 288.36)	3.31 (0.25, 145.98)	0.53 (0.10, 2.83)	PB+Chemotherapy	1.96 (0.38, 9.95)	0.92 (0.19, 4.61)	0.69 (0.14, 3.38)
0.31 (0.09, 1.06)	0.51 (0.15, 1.81)	0.49 (0.17, 1.52)	0.77 (0.19, 3.05)	0.39 (0.10, 1.70)	0.41 (0.10, 1.73)	0.13 (0.02, 0.99)	0.17 (0.03, 0.87)	0.31 (0.07, 1.46)	2.51 (0.20, 84.20)	3.41 (0.26, 159.65)	1.68 (0.12, 81.94)	0.28 (0.05, 1.46)	0.51 (0.10, 2.66)	BC+Chemotherapy	0.47 (0.10, 2.38)	0.35 (0.07, 1.92)
0.65 (0.21, 1.92)	1.08 (0.38, 2.94)	1.04 (0.32, 3.21)	1.61 (0.44, 5.43)	0.82 (0.23, 2.96)	0.86 (0.24, 2.94)	0.27 (0.04, 1.93)	0.35 (0.07, 1.74)	0.66 (0.23, 1.87)	5.35 (0.43, 179.22)	7.14 (0.56, 309.96)	3.56 (0.27, 151.04)	0.57 (0.11, 2.96)	1.08 (0.22, 5.19)	2.12 (0.42, 10.24)	AB+Chemotherapy	0.74 (0.17, 3.22)
0.87 (0.26, 2.79)	1.43 (0.48, 4.31)	1.39 (0.41, 4.55)	2.14 (0.57, 8.08)	1.10 (0.28, 4.29)	1.16 (0.29, 4.34)	0.36 (0.04, 2.71)	0.48 (0.09, 2.49)	0.88 (0.22, 3.57)	7.13 (0.55, 243.11)	9.59 (0.78, 405.95)	4.82 (0.35, 194.13)	0.77 (0.13, 4.34)	1.44 (0.30, 7.17)	2.83 (0.52, 14.76)	1.35 (0.31, 5.86)	CB+Chemotherapy

ORR

Chemotherapy	**1.73 (1.10, 2.84)**	**1.69 (1.02, 2.82)**	**2.57 (1.28, 5.22)**	1.29 (0.57, 3.08)	1.40 (0.52, 3.69)	0.53 (0.10, 2.89)	0.56 (0.17, 1.93)	1.03 (0.39, 2.82)	**9.35 (1.12, 258.29)**	**12.32 (1.24, 344.97)**	5.57 (0.56, 157.32)	0.91 (0.24, 3.41)	1.55 (0.48, 4.86)	3.43 (0.97, 12.43)	1.37 (0.43, 4.45)	1.19 (0.34, 4.42)
0.58 (0.35, 0.91)	Bevacizumab+Chemotherapy	0.98 (0.53, 1.74)	1.48 (0.67, 3.17)	0.74 (0.32, 1.77)	0.81 (0.35, 1.84)	0.31 (0.05, 1.76)	0.33 (0.09, 1.21)	0.59 (0.25, 1.46)	5.36 (0.61, 157.61)	7.07 (0.71, 201.05)	3.21 (0.29, 94.80)	0.52 (0.13, 2.12)	0.90 (0.25, 3.00)	1.99 (0.54, 7.20)	0.79 (0.27, 2.27)	0.69 (0.21, 2.27)
0.59 (0.35, 0.98)	1.02 (0.57, 1.90)	Cetuximab+Chemotherapy	1.52 (0.66, 3.49)	0.76 (0.30, 2.04)	0.83 (0.30, 2.29)	0.32 (0.05, 1.83)	0.34 (0.09, 1.23)	0.61 (0.22, 1.77)	5.55 (0.63, 156.72)	7.35 (0.70, 213.77)	3.31 (0.30, 96.58)	0.54 (0.13, 2.23)	0.92 (0.25, 3.22)	2.04 (0.64, 6.50)	0.81 (0.24, 2.71)	0.70 (0.19, 2.70)
0.39 (0.19, 0.78)	0.68 (0.32, 1.48)	0.66 (0.29, 1.53)	Panitumumab+Chemotherapy	0.50 (0.18, 1.50)	0.54 (0.18, 1.68)	0.21 (0.03, 1.32)	**0.22 (0.05, 0.88)**	0.40 (0.13, 1.31)	3.64 (0.39, 105.36)	4.81 (0.45, 140.34)	2.18 (0.18, 61.87)	0.35 (0.08, 1.55)	0.60 (0.16, 2.34)	1.34 (0.32, 5.50)	0.54 (0.14, 2.06)	0.46 (0.11, 1.88)
0.78 (0.33, 1.74)	1.35 (0.57, 3.10)	1.31 (0.49, 3.45)	1.99 (0.67, 5.69)	Sunitinib+Chemotherapy	1.09 (0.31, 3.50)	0.42 (0.06, 2.61)	0.45 (0.10, 1.84)	0.80 (0.23, 2.73)	7.34 (0.71, 218.28)	9.73 (0.81, 287.97)	4.37 (0.36, 137.10)	0.70 (0.14, 3.34)	1.21 (0.28, 5.02)	2.67 (0.59, 11.82)	1.07 (0.26, 4.14)	0.92 (0.21, 4.03)
0.72 (0.27, 1.91)	1.24 (0.54, 2.89)	1.21 (0.44, 3.38)	1.86 (0.60, 5.62)	0.92 (0.29, 3.23)	Cediranib+Chemotherapy	0.38 (0.05, 2.61)	0.41 (0.09, 1.89)	0.74 (0.23, 2.49)	6.67 (0.63, 215.65)	8.97 (0.77, 282.61)	4.03 (0.32, 123.41)	0.65 (0.13, 3.33)	1.12 (0.24, 4.84)	2.48 (0.52, 11.93)	0.97 (0.26, 3.94)	0.86 (0.20, 3.65)
1.87 (0.35, 10.44)	3.24 (0.57, 19.59)	3.14 (0.55, 18.64)	4.79 (0.76, 30.15)	2.41 (0.38, 15.87)	2.62 (0.38, 18.30)	Celecoxib+Chemotherapy	1.07 (0.14, 8.15)	1.91 (0.29, 13.78)	**18.64 (1.10, 646.04)**	**23.58 (1.47, 870.34)**	11.01 (0.56, 441.82)	1.66 (0.19, 14.68)	2.89 (0.37, 22.22)	6.42 (0.80, 54.39)	2.55 (0.33, 20.20)	2.23 (0.28, 18.26)
1.75 (0.52, 5.80)	3.03 (0.83, 11.33)	2.97 (0.81, 10.82)	4.50 (1.13, 18.35)	2.24 (0.54, 10.34)	2.45 (0.53, 11.67)	0.94 (0.12, 7.22)	Sorafenib+Chemotherapy	1.79 (0.40, 8.65)	**16.90 (1.42, 570.96)**	**21.97 (1.63, 735.30)**	10.07 (0.70, 322.41)	1.58 (0.28, 9.23)	2.75 (0.50, 14.90)	**6.03 (1.05, 34.63)**	2.38 (0.48, 13.52)	2.10 (0.37, 12.25)
0.97 (0.35, 2.59)	1.68 (0.68, 4.03)	1.64 (0.56, 4.64)	2.50 (0.76, 7.83)	1.25 (0.37, 4.29)	1.35 (0.40, 4.43)	0.52 (0.07, 3.50)	0.56 (0.12, 2.53)	Axitinib+Chemotherapy	9.23 (0.84, 288.08)	12.06 (0.98, 411.76)	5.65 (0.41, 167.28)	0.87 (0.17, 4.56)	1.52 (0.31, 6.75)	3.33 (0.67, 15.64)	1.33 (0.45, 3.82)	1.16 (0.26, 4.94)
0.11 (0.00, 0.89)	0.19 (0.01, 1.65)	0.18 (0.01, 1.59)	0.27 (0.01, 2.56)	0.14 (0.00, 1.40)	0.15 (0.00, 1.60)	0.05 (0.00, 0.91)	0.06 (0.00, 0.70)	0.11 (0.00, 1.19)	Trebananib+Chemotherapy	1.25 (0.03, 63.57)	0.60 (0.01, 30.90)	0.09 (0.00, 1.20)	0.16 (0.00, 1.85)	0.36 (0.01, 4.60)	0.14 (0.00, 1.76)	0.12 (0.00, 1.56)
0.08 (0.00, 0.80)	0.14 (0.00, 1.41)	0.14 (0.00, 1.42)	0.21 (0.01, 2.25)	0.10 (0.00, 1.23)	0.11 (0.00, 1.30)	0.04 (0.00, 0.68)	0.05 (0.00, 0.61)	0.08 (0.00, 1.02)	0.80 (0.02, 38.67)	Conatumumab+Chemotherapy	0.46 (0.01, 25.58)	**0.07 (0.00, 1.00)**	0.12 (0.00, 1.64)	0.27 (0.01, 3.72)	0.11 (0.00, 1.47)	0.09 (0.00, 1.37)
0.18 (0.01, 1.80)	0.31 (0.01, 3.47)	0.30 (0.01, 3.32)	0.46 (0.02, 5.41)	0.23 (0.01, 2.81)	0.25 (0.01, 3.14)	0.09 (0.00, 1.78)	0.10 (0.00, 1.43)	0.18 (0.01, 2.43)	1.66 (0.03, 115.62)	2.16 (0.04, 109.85)	Ganitumab+Chemotherapy	0.16 (0.00, 2.50)	0.27 (0.01, 3.85)	0.61 (0.02, 8.88)	0.24 (0.01, 3.41)	0.21 (0.01, 3.24)
1.10 (0.29, 4.14)	1.92 (0.47, 7.88)	1.87 (0.45, 7.68)	2.83 (0.64, 13.01)	1.42 (0.30, 7.04)	1.55 (0.30, 7.83)	0.60 (0.07, 5.17)	0.63 (0.11, 3.58)	1.15 (0.22, 5.91)	10.57 (0.83, 362.37)	13.85 (1.00, 448.48)	6.38 (0.40, 215.11)	Gefitinib+Chemotherapy	1.71 (0.30, 10.01)	3.86 (0.61, 23.95)	1.53 (0.26, 8.82)	1.31 (0.22, 8.44)
0.64 (0.21, 2.08)	1.11 (0.33, 4.05)	1.08 (0.31, 3.94)	1.65 (0.43, 6.44)	0.83 (0.20, 3.57)	0.89 (0.21, 4.16)	0.35 (0.05, 2.72)	0.36 (0.07, 2.01)	0.66 (0.15, 3.20)	6.18 (0.54, 203.00)	8.10 (0.61, 254.52)	3.68 (0.26, 119.49)	0.58 (0.10, 3.37)	PB+Chemotherapy	2.19 (0.41, 12.85)	0.88 (0.17, 4.70)	0.76 (0.14, 4.54)
0.29 (0.08, 1.03)	0.50 (0.14, 1.86)	0.49 (0.15, 1.56)	0.75 (0.18, 3.13)	0.37 (0.08, 1.70)	0.40 (0.08, 1.93)	0.16 (0.02, 1.25)	0.17 (0.03, 0.95)	0.30 (0.06, 1.50)	2.81 (0.22, 93.44)	3.67 (0.27, 129.16)	1.64 (0.11, 55.51)	0.26 (0.04, 1.64)	0.46 (0.08, 2.41)	BC+Chemotherapy	0.40 (0.08, 2.15)	0.35 (0.06, 1.98)
0.73 (0.22, 2.32)	1.26 (0.44, 3.66)	1.23 (0.37, 4.14)	1.87 (0.48, 7.02)	0.94 (0.24, 3.89)	1.03 (0.25, 3.89)	0.39 (0.05, 3.05)	0.42 (0.07, 2.10)	0.75 (0.26, 2.24)	7.06 (0.57, 236.77)	9.14 (0.68, 313.34)	4.17 (0.29, 132.51)	0.65 (0.11, 3.84)	1.13 (0.21, 5.96)	2.52 (0.46, 13.00)	AB+Chemotherapy	0.87 (0.18, 4.30)
0.84 (0.23, 2.92)	1.46 (0.44, 4.79)	1.43 (0.37, 5.27)	2.16 (0.53, 9.05)	1.08 (0.25, 4.65)	1.16 (0.27, 4.93)	0.45 (0.05, 3.61)	0.48 (0.08, 2.73)	0.86 (0.20, 3.81)	8.07 (0.64, 280.13)	10.57 (0.73, 359.53)	4.81 (0.31, 155.04)	0.76 (0.12, 4.58)	1.32 (0.22, 7.10)	2.89 (0.50, 16.57)	1.15 (0.23, 5.61)	CB+Chemotherapy

DCR

Chemotherapy	**2.55 (1.51, 4.54)**	1.28 (0.73, 2.29)	1.64 (0.79, 3.56)	0.92 (0.34, 2.54)	1.81 (0.63, 5.39)	0.32 (0.04, 2.26)	0.63 (0.13, 2.73)	1.41 (0.48, 4.29)	0.64 (0.16, 2.47)	1.39 (0.32, 5.67)	1.19 (0.30, 4.83)	0.79 (0.16, 3.73)	1.51 (0.43, 5.14)	2.13 (0.57, 8.41)	2.00 (0.56, 7.60)	2.27 (0.56, 9.65)
0.39 (0.22, 0.66)	Bevacizumab+Chemotherapy	**0.50 (0.25, 0.97)**	0.65 (0.28, 1.50)	**0.36 (0.13, 0.98)**	0.71 (0.28, 1.74)	**0.13 (0.01, 0.96)**	0.25 (0.05, 1.15)	0.55 (0.21, 1.39)	0.25 (0.06, 1.05)	0.54 (0.11, 2.56)	0.47 (0.10, 2.06)	0.31 (0.05, 1.62)	0.60 (0.14, 2.71)	0.84 (0.20, 3.33)	0.79 (0.23, 2.56)	0.89 (0.24, 3.29)
0.78 (0.44, 1.37)	2.00 (1.04, 4.00)	Cetuximab+Chemotherapy	1.29 (0.51, 3.21)	0.72 (0.24, 2.20)	1.42 (0.45, 4.31)	0.25 (0.03, 1.84)	0.50 (0.09, 2.42)	1.09 (0.36, 3.50)	0.50 (0.11, 2.16)	1.09 (0.23, 4.83)	0.93 (0.20, 4.17)	0.62 (0.11, 3.28)	1.18 (0.29, 4.55)	1.66 (0.49, 5.74)	1.58 (0.41, 6.10)	1.78 (0.42, 7.88)
0.61 (0.28, 1.27)	1.55 (0.67, 3.64)	0.78 (0.31, 1.95)	Panitumumab+Chemotherapy	0.56 (0.16, 1.87)	1.10 (0.32, 3.69)	0.20 (0.02, 1.51)	0.39 (0.07, 1.95)	0.86 (0.24, 3.05)	0.39 (0.08, 1.81)	0.84 (0.16, 4.02)	0.73 (0.15, 3.44)	0.48 (0.08, 2.76)	0.92 (0.21, 3.83)	1.29 (0.29, 5.91)	1.22 (0.29, 5.11)	1.38 (0.30, 6.57)
1.08 (0.39, 2.90)	2.76 (1.02, 7.98)	1.39 (0.45, 4.20)	1.79 (0.53, 6.07)	Sunitinib+Chemotherapy	1.95 (0.49, 7.88)	0.36 (0.03, 3.01)	0.68 (0.11, 3.85)	1.53 (0.39, 6.26)	0.71 (0.12, 3.80)	1.50 (0.24, 8.47)	1.29 (0.23, 6.85)	0.87 (0.13, 5.46)	1.64 (0.33, 7.85)	2.33 (0.45, 12.09)	2.20 (0.47, 10.52)	2.44 (0.48, 13.50)
0.55 (0.19, 1.59)	1.42 (0.58, 3.61)	0.71 (0.23, 2.23)	0.91 (0.27, 3.15)	0.51 (0.13, 2.03)	Cediranib+Chemotherapy	0.18 (0.02, 1.61)	0.35 (0.05, 2.10)	0.78 (0.21, 2.84)	0.35 (0.06, 1.96)	0.77 (0.13, 4.23)	0.66 (0.11, 3.79)	0.44 (0.06, 2.91)	0.84 (0.16, 4.14)	1.17 (0.13, 6.20)	1.12 (0.25, 4.81)	1.25 (0.26, 6.44)
3.10 (0.44, 24.92)	7.92 (1.05, 67.38)	3.97 (0.54, 34.27)	5.10 (0.66, 47.41)	2.82 (0.33, 30.11)	5.64 (0.62, 56.62)	Celecoxib+Chemotherapy	1.93 (0.16, 23.68)	4.38 (0.46, 45.51)	1.97 (0.18, 24.17)	4.29 (0.39, 52.77)	3.66 (0.34, 46.11)	2.45 (0.20, 31.17)	4.58 (0.47, 54.64)	6.65 (0.65, 81.54)	6.24 (0.61, 75.95)	6.91 (0.64, 88.11)
1.58 (0.37, 7.45)	4.02 (0.87, 21.57)	2.01 (0.41, 10.77)	2.59 (0.51, 14.60)	1.46 (0.26, 8.96)	2.85 (0.48, 19.87)	0.52 (0.04, 6.38)	Sorafenib+Chemotherapy	2.23 (0.37, 14.94)	1.00 (0.14, 7.82)	2.19 (0.29, 17.55)	1.89 (0.25, 15.49)	1.27 (0.15, 11.60)	2.38 (0.34, 17.95)	3.37 (0.46, 25.55)	3.20 (0.46, 24.89)	3.57 (0.48, 30.22)
0.71 (0.23, 2.08)	1.81 (0.72, 4.69)	0.91 (0.29, 2.81)	1.16 (0.33, 4.23)	0.65 (0.16, 2.58)	1.28 (0.35, 4.73)	0.23 (0.02, 2.19)	0.45 (0.07, 2.73)	Axitinib+Chemotherapy	0.46 (0.08, 2.49)	1.00 (0.16, 5.49)	0.84 (0.14, 4.86)	0.56 (0.08, 3.63)	1.08 (0.20, 5.73)	1.52 (0.28, 8.11)	1.42 (0.46, 4.46)	1.61 (0.33, 8.24)
1.56 (0.41, 6.20)	3.97 (0.96, 17.98)	2.00 (0.46, 9.08)	2.57 (0.55, 12.35)	1.42 (0.26, 8.09)	2.84 (0.52, 15.72)	0.51 (0.04, 5.59)	1.00 (0.13, 7.25)	2.18 (0.40, 13.21)	Trebananib+Chemotherapy	2.19 (0.30, 16.00)	1.85 (0.27, 13.34)	1.22 (0.15, 9.78)	2.37 (0.36, 14.87)	3.35 (0.51, 23.38)	3.11 (0.50, 20.87)	3.54 (0.53, 25.88)

0.72 (0.18, 3.11)	1.84 (0.42, 9.24)	0.92 (0.21, 4.32)	1.19 (0.25, 6.07)	0.67 (0.12, 4.11)	1.30 (0.23, 7.58)	0.23 (0.02, 2.56)	0.46 (0.06, 3.41)	1.00 (0.18, 6.23)	0.46 (0.06, 3.33)	Conatumumab+Chemotherapy	0.85 (0.12, 6.20)	0.58 (0.07, 4.67)	1.08 (0.16, 7.24)	1.54 (0.23, 11.45)	1.45 (0.22, 10.22)	1.63 (0.23, 12.64)
0.84 (0.21, 3.37)	2.14 (0.49, 9.91)	1.07 (0.24, 4.96)	1.38 (0.29, 6.78)	0.78 (0.15, 4.32)	1.51 (0.26, 8.90)	0.27 (0.02, 2.92)	0.53 (0.06, 3.98)	1.19 (0.21, 7.11)	0.54 (0.07, 3.76)	1.18 (0.16, 8.68)	Ganitumab+Chemotherapy	0.67 (0.08, 5.17)	1.25 (0.19, 8.17)	1.81 (0.26, 12.97)	1.69 (0.26, 11.41)	1.91 (0.27, 14.37)
1.26 (0.27, 6.28)	3.22 (0.62, 18.63)	1.62 (0.30, 8.92)	2.08 (0.36, 12.58)	1.16 (0.18, 7.99)	2.28 (0.34, 16.09)	0.41 (0.03, 5.09)	0.79 (0.09, 6.81)	1.79 (0.28, 12.57)	0.82 (0.10, 6.63)	1.74 (0.21, 14.48)	1.49 (0.19, 12.98)	Gefitinib+Chemotherapy	1.88 (0.25, 14.49)	2.68 (0.37, 22.20)	2.52 (0.35, 20.78)	2.85 (0.35, 25.68)
0.66 (0.19, 2.35)	1.67 (0.45, 7.08)	0.84 (0.22, 3.44)	1.08 (0.26, 4.80)	0.61 (0.13, 3.01)	1.19 (0.24, 6.27)	0.22 (0.02, 2.12)	0.42 (0.06, 2.97)	0.92 (0.17, 5.10)	0.42 (0.07, 2.80)	0.92 (0.14, 6.22)	0.80 (0.12, 5.34)	0.53 (0.07, 4.05)	PB+Chemotherapy	1.42 (0.23, 9.06)	1.33 (0.22, 8.52)	1.50 (0.23, 10.58)
0.47 (0.12, 1.76)	1.19 (0.30, 4.96)	0.60 (0.17, 2.02)	0.77 (0.17, 3.45)	0.43 (0.08, 2.24)	0.85 (0.16, 4.32)	0.15 (0.01, 1.54)	0.30 (0.04, 2.17)	0.66 (0.12, 3.57)	0.30 (0.04, 1.94)	0.65 (0.09, 4.32)	0.55 (0.08, 3.79)	0.37 (0.05, 2.71)	0.71 (0.11, 4.29)	BC+Chemotherapy	0.94 (0.15, 5.98)	1.05 (0.16, 7.28)
0.50 (0.13, 1.77)	1.27 (0.39, 4.28)	0.63 (0.16, 2.46)	0.82 (0.20, 3.50)	0.46 (0.10, 2.11)	0.89 (0.21, 3.98)	0.16 (0.01, 1.63)	0.31 (0.04, 2.19)	0.70 (0.22, 2.17)	0.32 (0.05, 2.00)	0.69 (0.10, 4.54)	0.59 (0.09, 3.91)	0.40 (0.05, 2.88)	0.75 (0.12, 4.54)	1.06 (0.17, 6.55)	AB+Chemotherapy	1.12 (0.19, 6.97)
0.44 (0.10, 1.77)	1.13 (0.30, 4.13)	0.56 (0.13, 2.40)	0.73 (0.15, 3.36)	0.41 (0.07, 2.08)	0.80 (0.16, 3.89)	0.14 (0.01, 1.57)	0.28 (0.03, 2.08)	0.62 (0.12, 3.00)	0.28 (0.04, 1.89)	0.61 (0.08, 4.28)	0.52 (0.07, 3.69)	0.35 (0.04, 2.89)	0.67 (0.09, 4.43)	0.95 (0.14, 6.06)	0.90 (0.14, 5.15)	CB+Chemotherapy

Notes: SD = stable disease; PD = progressive disease; CR = complete response; PR = partial response; ORR = overall response rate; DCR = disease control rate;PB=Panitumumab+Bevacizumab;BC=Brivanib+Cetuximab;AB=Axitinib+Bevacizumab;CB=Cetuximab+Bevacizumab;
